# Structural and signaling proteins in the Z-disk and their role in cardiomyopathies

**DOI:** 10.3389/fphys.2023.1143858

**Published:** 2023-03-02

**Authors:** Maya Noureddine, Katja Gehmlich

**Affiliations:** ^1^ Institute of Cardiovascular Sciences, College of Medical and Dental Sciences, University of Birmingham, Birmingham, United Kingdom; ^2^ Cardiovascular Medicine, Radcliffe Department of Medicine and British Heart Foundation Centre of Research Excellence Oxford, University of Oxford, Oxford, United Kingdom

**Keywords:** pathogenic variant, Z-disk protein, cardiomyopathy, alpha-actinin, filamin C (FLNC), titin (TTN), myopalladin (MYPN), desmin (DES)

## Abstract

The sarcomere is the smallest functional unit of muscle contraction. It is delineated by a protein-rich structure known as the Z-disk, alternating with M-bands. The Z-disk anchors the actin-rich thin filaments and plays a crucial role in maintaining the mechanical stability of the cardiac muscle. A multitude of proteins interact with each other at the Z-disk and they regulate the mechanical properties of the thin filaments. Over the past 2 decades, the role of the Z-disk in cardiac muscle contraction has been assessed widely, however, the impact of genetic variants in Z-disk proteins has still not been fully elucidated. This review discusses the various Z-disk proteins (alpha-actinin, filamin C, titin, muscle LIM protein, telethonin, myopalladin, nebulette, and nexilin) and Z-disk-associated proteins (desmin, and obscurin) and their role in cardiac structural stability and intracellular signaling. This review further explores how genetic variants of Z-disk proteins are linked to inherited cardiac conditions termed cardiomyopathies.

## 1 Introduction

Inherited cardiomyopathies are a group of genetic diseases affecting the heart muscle, which are caused by pathogenic variants in different cardiac proteins. They are a major cause of mortality and morbidity worldwide (Sabater-Molina et al., 2018). Most cardiomyopathies are inherited in an autosomal-dominant pattern (Ehsan et al., 2017) and are classified based on the dominant functional or morphological changes in the myocardium. The different types include hypertrophic cardiomyopathy (HCM), dilated cardiomyopathy (DCM), arrhythmogenic cardiomyopathy (ACM), restrictive cardiomyopathy (RCM) and left ventricular non-compaction (LVNC) (Ehsan et al., 2017).

HCM is one of the most common cardiomyopathies with a prevalence of 1:500, and is often associated with sudden cardiac death (Mendes de Almeida et al., 2017). This disease is characterized by left ventricular hypertrophy and a non-dilated left ventricle with diastolic dysfunction, but with preserved or increased ejection fraction (Marian and Braunwald, 2017). At the cellular level, cardiomyocyte disarray, cellular hypertrophy, and interstitial fibrosis are hallmarks of the disease (Marian and Braunwald, 2017).

DCM is characterized by increased left ventricular chamber size and systolic dysfunction leading to progressive heart failure (Begay et al., 2016). It is known as one of the leading causes of cardiovascular mortality and heart failure, and patients with this disease often require heart transplantation (Zhao et al., 2015). Estimates of prevalence vary between 1:250 and 1:2500 (Rakar et al., 1997; Hershberger et al., 2013).

ACM is pathologically characterized by fibrofatty myocardial replacement and clinically by ventricular electrical instability, which predisposes patients to life-threatening ventricular arrhythmias and sudden cardiac death (Corrado and Zorzi, 2018). It is prevalence is reported to be 1:2000 (Groeneweg et al., 2014).

RCM is a rare, fatal myocardial disease caused by diastolic dysfunction, i.e., increased myocardial stiffness and impaired ventricular filling, with usually preserved ventricular dimensions (Muchtar et al., 2017).

LVNC can occur in isolation or as part of other cardiomyopathies. It is an abnormally prominent trabeculation of the left ventricle, usually diagnosed by imaging (echocardiography or cardiac magnet resonance imaging) (Srivastava et al., 2022).

**FIGURE 1:**
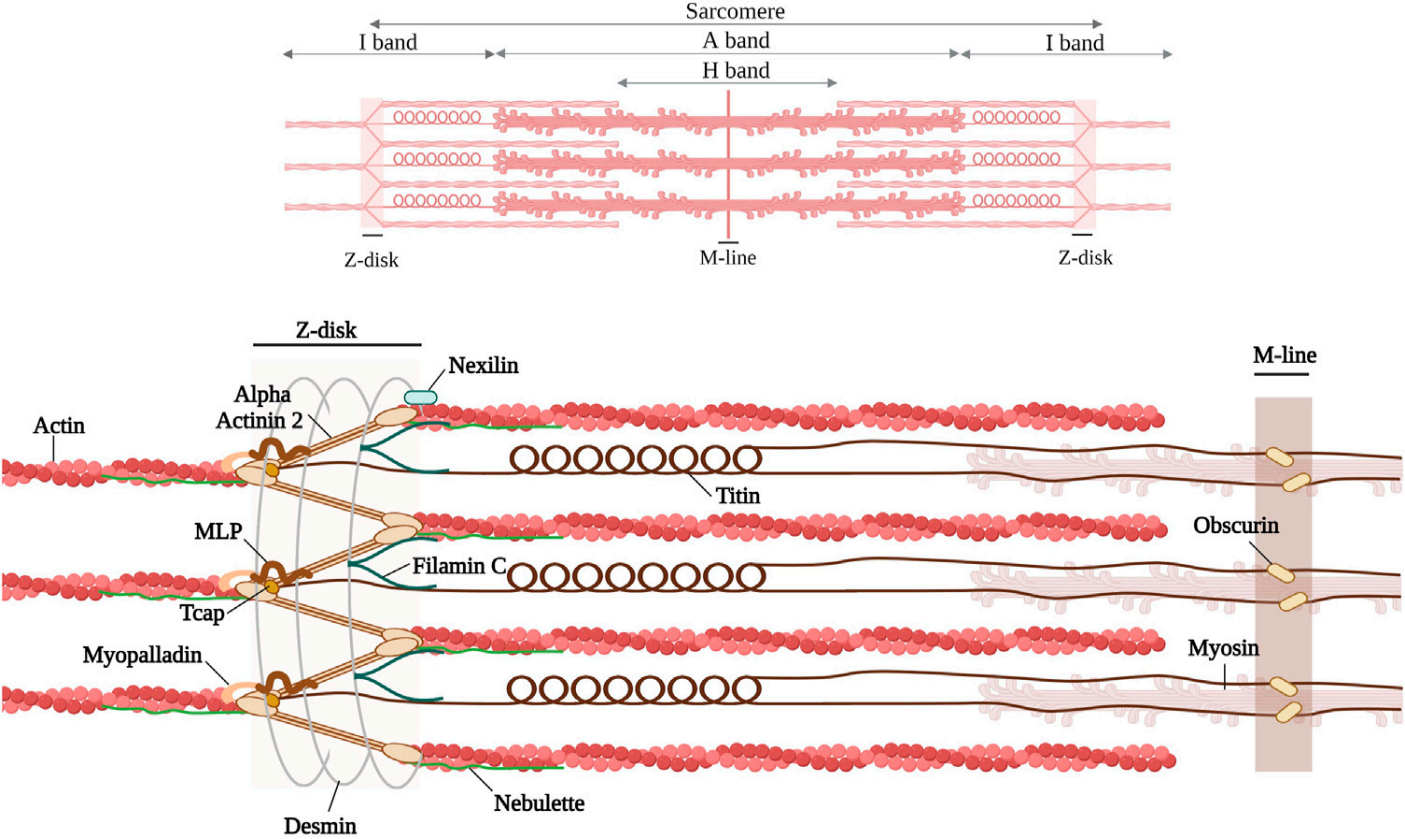
Schematic representation of the discussed Z-disk proteins (alpha-actinin 2, Filamin C, Titin, muscle LIM protein, telethonin, myopalladin, nebulette, nexilin) and Z-disk associated proteins (desmin and obscurin). Created with BioRender.com. Adapted from (Pyle and Solaro, 2004; Mukund and Subramaniam, 2020).

Inherited cardiomyopathies result in structural and contractile alterations in the cardiac muscle. Structurally, myocardial filaments are composed of sarcomeres, the basic contractile units. At the boundaries of the sarcomeres, a multiprotein complex termed the Z-disk tethers actin-rich thin filaments. The Z-disk plays an integral role in the structure and function of striated muscle; it provides, together with the M-band tethering thick filaments, the structural integrity of the sarcomeres. Moreover, the Z-disk also functions as a signaling hub by converting biomechanical stress into biochemical signals that are important for adaptation to mechanical stress (Frank and Frey, 2011; Filomena et al., 2021). Furthermore, there are numerous proteins that interact with each other at the Z-disk (see Figure 1). The Z-disk proteins also connect the contractile apparatus to the cytoskeleton and the extracellular matrix (Clark et al., 2002; Zhang et al., 2007). Pathogenic variants in these structural or regulatory proteins can in turn lead to structural and functional impairment at the Z-disk, resulting in cardiomyopathies or other pathologies, e.g., skeletal muscle diseases (Avnika et al., 2012; Wadmore et al., 2021).

This review focuses on various cardiac Z-disk proteins (alpha-actinin, filamin C, titin muscle LIM protein, telethonin, myopalladin, nebulette, nexilin), all known to bind actin and to be associated with cardiomyopathies, and how genetic variants in these proteins are associated with different cardiomyopathies. We have further included two and Z-disk-associated proteins, desmin and obscurin (Figure 1). Due to space constraints we cannot discuss all components of the Z-disk, e.g., for alpha-beta crystallin, myotilin, alpha-actinin associated LIM protein (ALP), Z-band Alternative Spliced PDZ motif (ZASP), or filamin-, α-actinin-, and telethonin-binding protein of the Z-disk (FATZ), we refer to excellent reviews (Faulkner et al., 2001; Knöll et al., 2011a; Wadmore et al., 2021).

The evaluation of genetic variants and their role in cardiomyopathies is complex and multidimensional as outlaid in the American College of Medical Genetics and Genomics guidelines (Richards et al., 2015). In addition, with the advancement of high-throughput sequencing techniques the understanding of genetic variation in normal populations has evolved. This means that genetic variants or entire genes previously implicated in a disease, based on Sanger sequencing of small cohorts, may not be considered to contribute significantly to the disease today, as was demonstrated for HCM (Ingles et al., 2019) and DCM (Jordan et al., 2021).

A recent large scale genetic study has suggested a threshold of minor allelic frequency (MAF) lower than 1 × 10^−4^ in the cohort database of the Exome Aggregation Consortium for a missense variant to be considered pathogenic. This is based in the fact that the most common HCM-associated *MYPBC3* missense variant p. Arg502Trp was only found at MAF of 2.5 × 10^−5^ in the database, hence no other cardiomyopathy variant is likely to be more common. While this review cannot re-evaluate all reported genetic variants in the genes we discuss according to the American College of Medical Genetics and Genomics guidelines (Richards et al., 2015), we have applied the same MAF cutoff and only discuss variants with MAF <1 × 10^−4^, using the Genome Aggregation Database (GnomAD, which is the largest cohort database available at the moment). This frequency threshold will help to better distinguish rare pathogenic variants in Z-disk protein from more common variants, which are unlikely to cause monogenic disease (Fahed et al., 2020).

## 2 Z-disc proteins and their association with cardiomyopathies

### 2.1 Alpha-actinin

#### 2.1.1 Structure and function of alpha-actinin

**FIGURE 2:**
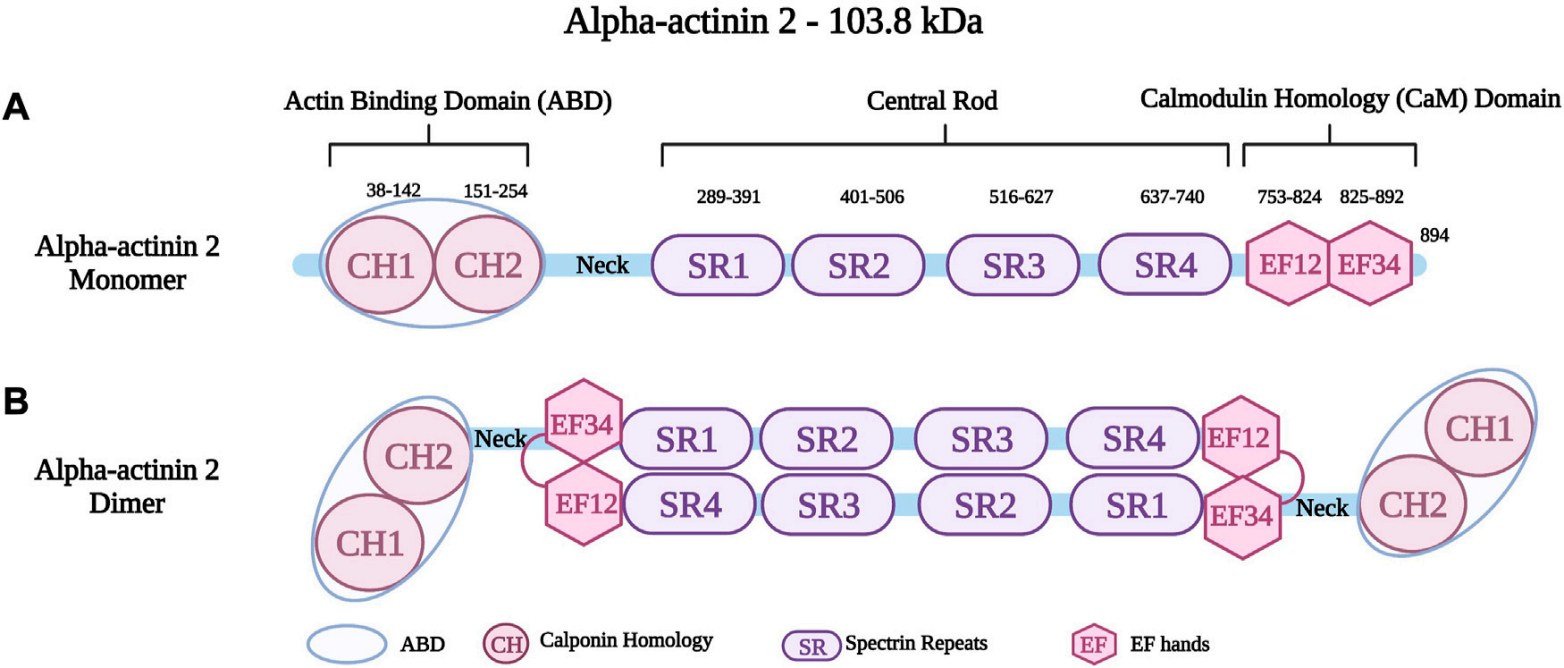
**(A)** Schematic representation of alpha-actinin two monomer with different residues in the Actin Binding Domain (ABD), Spectrin repeats (SR) and EF hands (EF). **(B)** Schematic representation of alpha-actinin two dimer showing the interaction between the neck region and the EF hands. Created with BioRender.com. Adapted from (Haywood et al., 2016).

Alpha-actinin is a protein with a molecular weight of 94 kDa–104 kDa. It forms antiparallel, actin-crosslinking homodimers (Hein et al., 2009). Alpha-actinin belongs to a highly conserved family of actin-binding proteins, the spectrin superfamily (Dixson et al., 2003; Sjöblom et al., 2008; Chiu et al., 2010). This family consists of short and long actin cross-linkers (such as the alpha-actinins and the alpha- and beta-spectrin) as well as dystrophin and utrophin, which are monomeric actin filament binding proteins and membrane adaptors (Ylänne et al., 2001; Dixson et al., 2003; Sjöblom et al., 2008; Foley and Young, 2014). The name of the spectrin superfamily derives from the presence of specific spectrin repeats (Foley and Young, 2014). All members of this family, including alpha-actinin have an amino-terminal actin-binding domain (ABD), consisting of two consecutive calponin homology domains (CH1 and CH2) (Figure 2A) (Sjöblom et al., 2008). The adjacent alpha-helical neck region connects the ABD with a central rod consisting of spectrin repeats with the number of repeats determining the flexibility and the nature of actin-binding properties (Ylänne et al., 2001; Sjöblom et al., 2008). The rod domain of alpha-actinin monomers comprises of four spectrin repeats, which interact to form antiparallel dimers (Figure 2B) that act on cross-linking actin filaments (Clark et al., 2002). In the carboxy-terminal region, there is a calmodulin homology domain (Critchley, 2000) that consists of two pairs of EF-hand motifs (EF1/2 and EF 3/4) (Sjöblom et al., 2008; Ribeiro Ede et al., 2014; Lindholm et al., 2021). EFs act on distinguishing between the four isoforms of alpha-actinin. The non-muscle isoforms (*ACTN1* and *ACTN4*) bind calcium through their EFs, whereas the EFs of the muscle isoforms (*ACTN2* and *ACTN3*) have lost their ability to bind calcium (Sjöblom et al., 2008; Hein et al., 2009; Chiu et al., 2010).

Alpha-actinin 2 (encoded by *ACTN2* on chromosome 1q43), is a major isoform localized at the Z-disk of the sarcomere (Hein et al., 2009; Prondzynski et al., 2019). Like all alpha-actinins, it forms antiparallel dimers. An intramolecular interaction of the neck region with EF3/4 keeps the dimer in a closed confirmation, which can be opened upon phospholipid binding (Ribeiro Ede et al., 2014). It is known to anchor and cross-link actin and titin filaments from adjacent sarcomeres (Ylänne et al., 2001; Chiu et al., 2010; Lindholm et al., 2021). This crucial protein stabilizes the contractile muscle apparatus by forming a lattice-like structure between titin and actin filaments (Sjöblom et al., 2008; Good et al., 2020). Alpha-actinin two further plays a crucial role in the organization of thin filaments by linking the cytoskeleton to different transmembrane proteins (Sjöblom et al., 2008). In addition to the role of alpha-actinin two in the structural integrity of the sarcomere, it also has important cellular roles in regulating the transactivation activity of various receptors, such as glucocorticoid receptor interacting protein-1 (GRIP1) (Huang et al., 2004). Moreover, it also regulates ion channels such as potassium channels (Kv1.5 and Kv1.4) (Cukovic et al., 2001) and the Ca^2+^ activated K^+^ channel (SK2 channel) (Lu et al., 2009). Other roles include connecting the sarcomeric cytoskeleton to various transmembrane proteins (Otey and Carpen, 2004). Global inactivation of alpha-actinin two in mice is not compatible with life, but a cardiac specific mosaic inactivation suggests a crucial role of the protein for cardimyocyte maturation *via* serum response factor signaling (Guo et al., 2021). Moreover, a role of alpha-actinin two in regulating cardiac mitochondrial function has been proposed recently (Zech et al., 2022), in part by acting as a scaffold for mitochondrial messenger RNAs *via* binding to RNA-binding proteins (Ladha et al., 2020).

#### 2.1.2 Alpha-actinin two and cardiomyopathies

Several studies have shown an association between pathogenic variants in *ACTN2* and various cardiac manifestations, including HCM, DCM, and ACM. A study by Prondzynski et al. identified a rare variant in *ACTN2* (p.Thr247Met) in one HCM patient with left ventricular hypertrophy, outflow tract obstruction, and atrial fibrillation (Prondzynski et al., 2019). Human induced pluripotent stem cells (iPSCs) were reprogrammed from the patient, and subsequently, differentiated into iPSC cardiomyocytes (iPSC-CMs), and engineered heart tissues. The results revealed several features of HCM including myofibrillar disarray, cardiomyocyte hypertrophy, impaired relaxation, hypercontractility, increased myofilament calcium sensitivity, and prolonged action potential duration (Prondzynski et al., 2019). A recent study by Zech et al. involved used iPSC-CMs heterozygous and homozygous for this *ACTN2* variant (p.Thr247Met) and showed multinucleation, cardiomyocyte hypertrophy, and myofibrillar disarray in the cellular models (Zech et al., 2022).

A study by Chiu et al. involved 23 patients with HCM and the results identified an *ACTN2* missense variant (p.Ala119Thr). In addition, the overexpression of this variant resulted in a significant increase in RNA markers for cardiomyocyte hypertrophy (Chiu et al., 2010). Another study examined a large four-generation family with autosomal dominant cardiomyopathic features including mid-apical HCM with marked bilateral dilatation, LVNC, and early onset of atrial fibrillation and atrioventricular block. The study identified a pathogenic variant (p.Met228Thr) in *ACTN2* (Girolami et al., 2014). In another study by Theis et al., two *ACTN2* genetic variants (p.Gly111Val and p. Thr495Met) were mapped from two patients with HCM, and a pathology report showed marked cardiomyocyte hypertrophy, focal myocyte disarray, endocardial fibrosis, and interstitial fibrosis (Theis et al., 2006). A study by Haywood et al. functionally characterized two *ACTN2* variants (p.Gly111Val and p. Ala119Thr), located in the actin-binding domain. These variants impaired alpha-actinin two function by decreasing F-actin binding affinity and altering Z-disk localization. This contributes to the disease phenotype of HCM (Haywood et al., 2016).

Another study by Lindholm et al. derived iPSC-CMs from a patient presenting with tachypnea and reduced ejection fraction. This patient was found to carry a rare *ACTN2* truncating variant (p.Gln860Ter). The iPSC-CMs were hypertrophic, exhibited structural disarray of the sarcomere, and impaired contractility. Moreover, this variant resulted in a loss in the carboxy-terminus of alpha-actinin 2, hence leading to disruption of the interaction with two sarcolemma-associated proteins: alpha-actinin 1 (*ACTN1*) and Gap junction alpha-1 protein (*GJA1*), which may explain the clinical arrhythmic and relaxation defects (Lindholm et al., 2021).

A study by Fan et al. involved a Chinese family with DCM, and ventricular tachycardia. This family was found to have a likely pathogenic variant in *ACTN2* (p.Leu320Arg) which was identified by whole-genome sequencing in four out of 12 affected family members (Fan et al., 2019). In another study, a *ACTN2* variant (p.Ala119Thr) was detected in an Australian family that exhibited a cardiac phenotype of DCM as well as idiopathic ventricular fibrillation, LVNC, and sudden death. Using exome sequencing, this variant was identified as responsible for the cardiac phenotype that the family exhibited (Bagnall et al., 2014). In a study by Good et al., a clinical investigation was performed on a family with left-dominant ACM. The *ACTN2* missense variant (p.Tyr473Cys) was identified in four family members and was considered causative of familial left dominant ACM (Good et al., 2020).

In conclusion, several studies have localized *ACTN2* pathogenic variants in different regions of alpha-actinin 2 (Supplementary Table S1). Studies have best documented the association of *ACTN2* pathogenic variants and HCM, albeit as rare causes of the disease (Theis et al., 2006; Chiu et al., 2010; Haywood et al., 2016). In contrast, the role of *ACTN2* variants in both DCM and ACM is less well understood.

### 2.2 Filamin C

#### 2.2.1 Structure and function of filamin C

**FIGURE 3:**
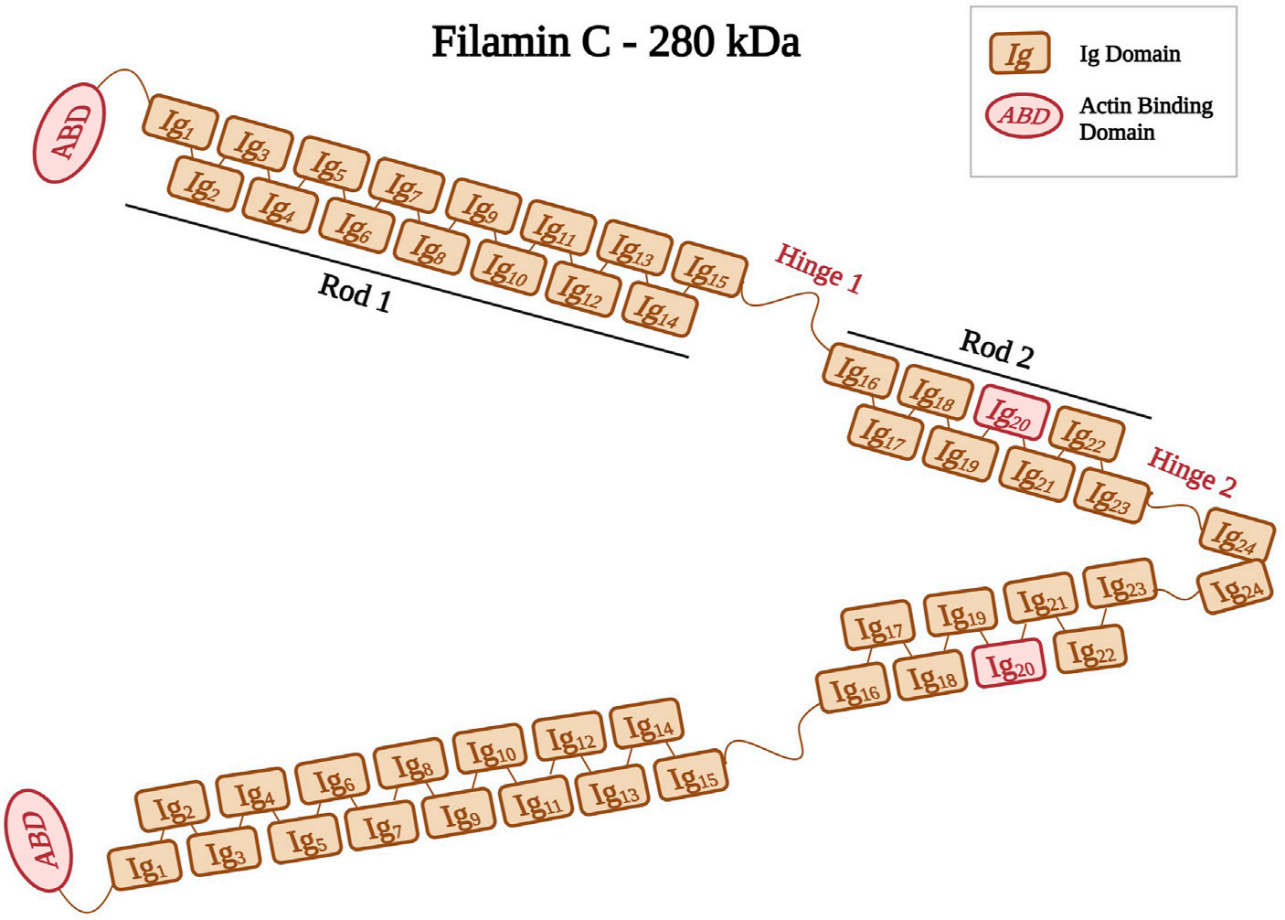
Schematic representation of filamin C shown as a dimer with different Ig domains forming both Rods (Rod1, Ig1 to Ig15; Rod2, Ig16 to Ig23). Positions of hinges one and two are also indicated. Ig20 is highlighted in red because of its unique 80 amino acid insertion. Created with BioRender.com.

Filamins are elongated dimers (Stossel et al., 2001) that cross-link and bind to F-actin filaments (Stossel et al., 2001; Popowicz et al., 2006; Leber et al., 2016). The filamin dimer is also called actin-binding protein-280 (ABP-280) (van der Ven et al., 2000) and it consists of two 280 kDa subunits (Himmel et al., 2003). Filamins have an ABD at their amino-terminus, which consists of two calponin homology domains (CH1 and CH2) (Bañuelos et al., 1998; van der Flier and Sonnenberg, 2001; Himmel et al., 2003). The ABD is followed by a rod domain consisting of 24 immunoglobulin-like (Ig) repeats structured into two rod regions (Rod one consists of Ig_1_ to Ig_15_ and Rod2 consists of Ig_16_ to Ig_23_) interrupted by two hinges (hinge one and 2) (Figure 3) (van der Flier and Sonnenberg, 2001; Pudas et al., 2005; Leber et al., 2016). The last Ig domain (Ig_24_) is present at the terminal end, and it allows filamins to dimerize. This structural organization allow filamins to adopt a flexible V-shaped structure that is essential for their function (Feng and Walsh, 2004; Zhou et al., 2007).

In addition, filamins have numerous binding partners, including intracellular signaling molecules, integrin receptors (β1D-integrin) (Loo et al., 1998), δ- and γ-sarcoglycans (Thompson et al., 2000), ion channels, and transcription factors (Himmel et al., 2003; Zhou et al., 2007; Nakamura et al., 2011). The interaction of filamins with different binding partners affects cellular activities, such as cytoskeletal organization, cellular motility, and differentiation (Razinia et al., 2012). Filamins also play an important role in maintaining the stability of the sarcomere and transmitting and responding to the mechanical forces acting on the F-actin network (Stossel et al., 2001; Feng and Walsh, 2004; Razinia et al., 2012).

The filamin family includes three members, filamin A, filamin B, and filamin C (Feng and Walsh, 2004). Filamin A and B are widely expressed, whereas filamin C is expressed predominantly in both skeletal and cardiac muscles (Stossel et al., 2001; Zhou et al., 2007). Filamin C is located at the Z-disks, costameres, and intercalated disks (Brodehl et al., 2017). Its gene *FLNC* is mapped to the chromosomal region 7q32–q35 (Gariboldi et al., 1994; Kley et al., 2012). Filamin C acts on anchoring and cross-linking actin filaments into a meshwork (van der Ven et al., 2000; Himmel et al., 2003; Begay et al., 2016). It plays an important role in the Z-disk assembly (van der Ven et al., 2006), where it anchors various proteins, such as myopodin, and myotilin (Thompson et al., 2000), to the actin cytoskeleton (van der Flier and Sonnenberg, 2001). Filamin C has a unique 80 amino acid insertion in its 20th Ig domain (Ig_20_) that mediates binding to various ligands and muscle proteins, such as Xin actin-binding repeat-containing proteins (XIRP1 and 2) (Molt et al., 2014; Mao and Nakamura, 2020). This interaction between Filamin C and XIRPs allows the stabilization of actin filaments by regulating cell adhesion dynamics (Seppälä et al., 2015).

The importance of filamin C for cardiac integrity is highlighted in two independent animal studies: cardiac-specific, and inducible *FLNC* knockout mouse models led to rapid DCM and heart failure. The study by Powers et al. demonstrated rapid onset DCM, with significant cardiomyocyte lengthening without a change in width. The mice were also found to have disrupted Z-disk myofilament organization (Powers et al., 2022). In another study by Zhuo et al., death of an inducible, cardiac specific *FLNC* knockout mice model occurred within 1 week of ablation of the protein due to heart failure. Z-disk, costameres and intercalated disc structures were affected in the hearts (Zhou et al., 2020).

#### 2.2.2 Filamin C and cardiomyopathies

Pathogenic variants in *FLNC* can affect sarcomere structure and function, leading to cardiac dysfunction and cardiomyopathies (Fürst et al., 2013). Missense variants in *FLNC* have been identified as novel causes of HCM. In a study by Valdés-Mas et al., whole-exome sequencing (WES) was performed in 92 HCM cases, and the following *FLNC* variants were identified (p.Val123Ala, p. Ala1539Thr, and p. Arg2133His). Patients with these *FLNC* variants show marked abnormalities in sarcomeres and large aggregates of filamin C (Valdés-Mas et al., 2014).

Gomez et al. performed next-generation sequencing (NGS) in 448 patients with HCM, and 20 different *FLNC* variants were identified (19 missense and one non-sense), suggesting that *FLNC* pathogenic variants are an important cause of this cardiomyopathy (Gómez et al., 2017).

A study by Ader et al. included sequencing of 1150 patients (among them 700 with HCM and 300 with DCM), and multiple pathogenic *FLNC* variants were identified in 28 patients (See Supplementary Table S2) (Ader et al., 2019). They suggest that truncation variants in *FLNC* are prevalent in DCM, while missense or small in frame deletions are seen in other types of cardiomyopathies.

In a study by Chanavat et al., NGS was performed on a cohort of 90 patients, with two variants identified in HCM cases (p.Arg2045GLn and p. Arg2018His) and one variant identified in a DCM case (p.Tyr1840Ter) (Chanavat et al., 2016). A study by Begay et al., a *FLNC* variant (p.Gly1891Val) was identified by WES in two Italian families with DCM and a clinical presentation of supraventricular tachycardia and atrial fibrillation (Begay et al., 2016).

In a further study, 319 families with DCM from the United States and Europe were examined using WES and NGS. Six different *FLNC* truncation variants were identified where 11 of the 13 truncation carriers had ventricular arrhythmias or sudden cardiac death (Begay et al., 2018). In a study by Augusto et al., seven DCM patients out of 89 had different *FLNC* truncations and showed abnormalities in left ventricular wall motion, left ventricular impairment, and non-sustained ventricular tachycardia (see Supplementary Table S2) (Augusto et al., 2020).

In addition, *FLNC* variants have been associated with the pathogenesis of other cardiomyopathies, e.g., ACM. In a study by Ortiz-Genga et al., NGS was used on 2,877 patients with inherited cardiovascular diseases including (1078 with HCM, 508 with DCM, 219 with ACM, and several other diseases). 23 *FLNC* truncating variants were identified in 28 patients (20 with DCM, seven with ACM, and one with RCM) (Ortiz-Genga et al., 2016).

Another study by Celeghin et al. included 270 participants, 12 *FLNC* variants (five missense and seven truncating ones) were mapped in 18 patients with ACM (Celeghin et al., 2022). In another study, WES of 120 gene-elusive ACM index cases identified four truncating *FLNC* variants (Hall et al., 2020). Moreover, Brun et al. (2020) also tested 156 patients with ACM and detected two *FLNC* truncations (p.Glu2189Ter and p. Asp2703ThrfsTer69) (Brun et al., 2020).

Some studies also investigated the association between *FLNC* variants and RCM. A study by Brodehl et al. tested 123 patients and revealed two novel *FLNC* missense variants (p.Ser1624Leu and p. Ile2160Phe) (Brodehl et al., 2016). Another study also identified two *FLNC* variants (p.Pro2298Leu and p. Tyr2563Cys) in two families with RCM (Schubert et al., 2018). In a further study by Kiselev et al., a cohort of 28 patients with early-onset RCM was screened and the *FLNC* variant (p.Ala1186Val) was identified in three patients and (p.Ala1183Leu) in one patient (Kiselev et al., 2018).

A functional study by Tucker et al. involved eight patients with RCM and WES identified a novel *FLNC* missense variant (p.Val2297Met). iPSC-CMs with the variant displayed decreased contractility compared to the wild type, thus suggesting a defect in the contractile apparatus (Tucker et al., 2017). Another study by Agarwal et al. detected the formation of filamin C aggregates when a heterozygous in-frame deletion of *FLNC* (p.Val1668_Gly1674del) was introduced into iPSC-CMs. Other observations included disassembly of the sarcomere structure and a reduction in cardiomyocyte contractility (Agarwal et al., 2021). Further, Chen et al. generated iPSC lines from two DCM patients with two *FLNC* truncating variants (p.Gly1891Valfs61Ter, and p. Glu2189Ter). These iPSC-CMs displayed impaired contraction evidenced by a weaker contraction and slower relaxation. They also exhibited spontaneous arrhythmia and iPSC-CMs with the variants were found to have a lower expression of filamin C protein and mRNA (Chen et al., 2022).

In conclusion, *FLNC* variants are associated with different cardiomyopathies (see Supplementary Table S2). It emerges that truncating variants in *FLNC* are associated with DCM and ACM without overt skeletal muscle pathologies. Currently it is enigmatic why other truncating variants in the same gene cause predominantly skeletal muscle disease (Selcen and Carpén, 2008). Moreover, missense variants in *FLNC* have been associated with HCM and RCM, however assigning causality to individual variants is challenging as missense variants in *FLNC* also occur frequently in normal cohorts.

### 2.3 Titin

#### 2.3.1 Structure and function of titin

Titin protein, also known as connectin, is a giant elastic filament that spans half the sarcomere (Bang et al., 2001a). It is the third most abundant muscle protein (after myosin and actin) in the mammalian organisms and has a molecular weight of 3 MDa (Maruyama, 1997; Granzier and Labeit, 2002; Au, 2004). The titin gene (*TTN*) is located on chromosome 2q31, which contains 363 exons (Bang et al., 2001a) and codes for 26,926 amino acids (Labeit and Kolmerer, 1995). Titin extends over half of the sarcomere, and it connects the thin filaments at the Z-disk with its amino-terminus to the thick filaments at the M-band with its C-terminus (Fürst et al., 1988; Maruyama, 1997; Au, 2004). The I-band region of titin is responsible for the elasticity of muscle tissues (Chanavat et al., 2016). Titin’s A-band region helps to stabilize thick filaments (reviewed in (Chauveau et al., 2014; Gigli et al., 2016)).

Titin exons undergo extensive alternative splicing thus producing two titin isoforms (N2B and N2BA) that are expressed in the myocardium. The two isoforms span the sarcomere Z-disk to M-band but differ primarily in the I-band resulting in different sizes. N2BA isoform consists of both the N2A and N2B segments while the N2B isoform consist of a smaller PEVK segment, fewer Ig domains and it lacks the N2A segment (Granzier and Labeit, 2004; Nagueh et al., 2004; Roberts et al., 2015).

Titin has several roles including the generation of the passive force (Kollár et al., 2010), maintaining sarcomeric alignment and regulating myosin filament assembly (Whiting et al., 1989; Labeit and Kolmerer, 1995). By binding a multitude of signaling proteins, titin also plays a central role in integrating mechanical and hypertrophic signals from and to the sarcomere (Granzier et al., 2009; Kollár et al., 2010).

Global inactivation of titin in animals is not compatible with life, highlighting the crucial function of the protein for muscle integrity. However, spontaneous natural mutations or targeted engineered genetic variants, or deletions of titin regions, in fish or rodents, have shed light on important functions of the protein and are reviewed in (Marcello et al., 2022).

#### 2.3.2 Titin Z-disk portion

**FIGURE 4:**
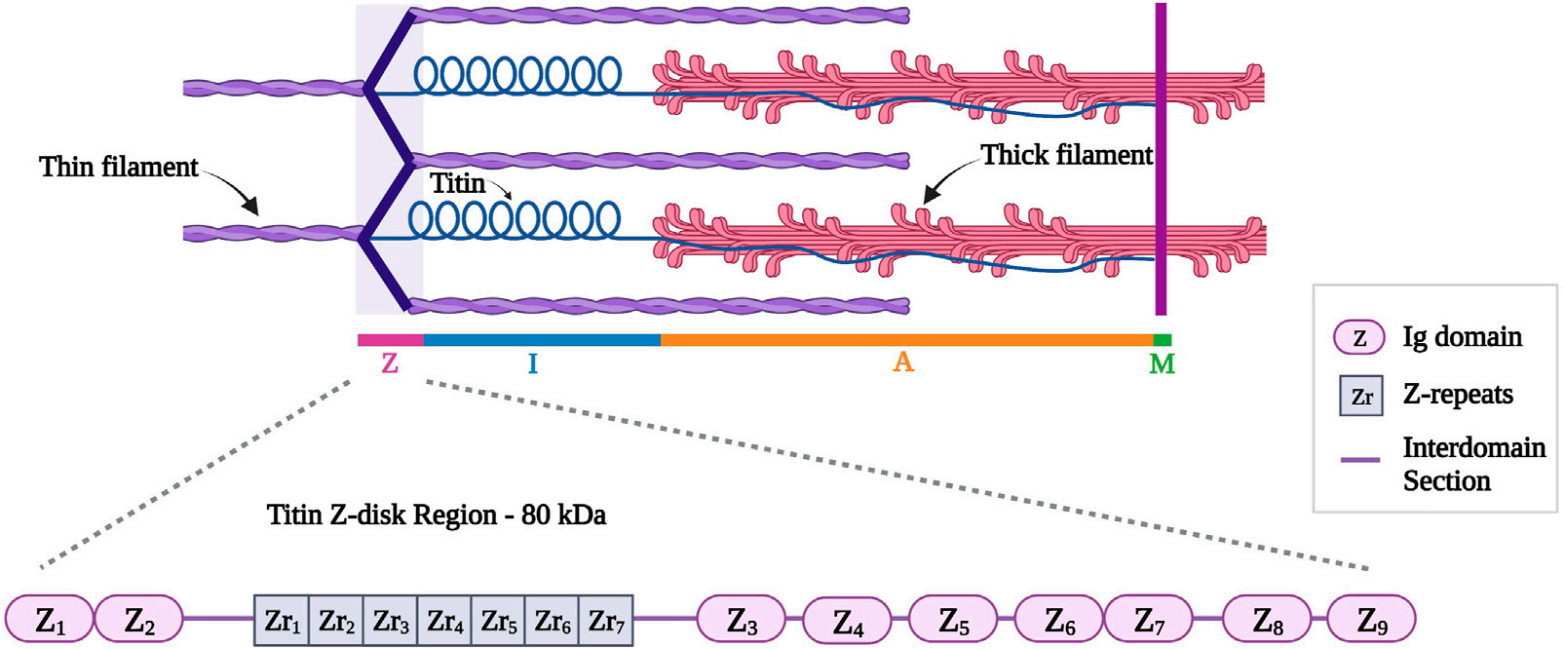
Schematic representation of titin (in blue) different regions with a detailed representation of titin Z-disk region with different Ig domains (Z1 to Z9) and Z-repeats (Zr1 to Zr7). Created with BioRender.com.

This review will focus on the Z-disk portion of titin (first 15 exons, approximately 80 kDa). Titin Z-disk portion is composed of ∼900 residues and it consists of consecutive immunoglobulin (Ig)-like domains and 45-residue repeating modules (called Z-repeats or Zr) (Figure 4) (Gautel et al., 1996; Tonino et al., 2019). The Z-repeats vary in number according to the muscle type (Luther and Squire, 2002). The first two Ig domains (termed Z1 and Z2) in titin’s amino-terminus bind telethonin (also known as T-cap). This interaction is the strongest, non-covalent interaction described between two proteins (Bertz et al., 2009) and it allows anti-parallel alignment of adjacent titin molecules within the Z-disk (Gregorio et al., 1998; Young et al., 1998; Zou et al., 2006). In addition, the fifth Z-repeat (Zr5) of titin has been found to bind the C-terminus of alpha-actinin (Ohtsuka et al., 1997). Several studies discussed the interaction between titin and alpha-actinin to take place between the seventh Z-repeat (Zr7) of titin and the EF hands (EF34) of alpha-actinin (Sorimachi et al., 1997; Young et al., 1998; Atkinson et al., 2000; Joseph et al., 2001; Haywood et al., 2016).

#### 2.3.3 Titin and cardiomyopathies

Several studies have shown that pathogenic variants in *TTN* are associated with DCM. The role of truncating variants in *TTN* (TTNtv) is well established in DCM (Herman et al., 2012), where a large study found that TTNtv associated with DCM are found in the exons with high cardiac expression (Roberts et al., 2015). In addition, the position of the variant within the gene plays an important role. For instance, the association between *TTN* truncating variants and DCM in the A-band portion has a much higher odds ratio (odds ratio 49.8) than in the Z-disk portion (odd ratio 5.3) (Schafer et al., 2017).

In contrast, truncating variants in *TTN* are not associated with HCM: In a study by Zhang et al., WES was performed on 529 Chinese patients with HCM and 307 healthy controls. They identified 14 *TTN* truncating variants (six non-sense, two frameshift and six splice site variants) in 13 HCM patients (13 of 529 [2.5%]) and eight controls (8 of 307 [2.6%]). Thus, no significant difference was observed between the HCM patients and the controls carrying the *TTN* variants (Zhang et al., 2017).

In a study by Taylor et al., 38 families with ACM were sequenced and eight TTN missense variants were detected in seven families. One of the variants (p.Thr2896Ile) is located at the 10th Ig domain and was shown to reduce the structural stability of this domain (Taylor et al., 2011).

Due to the high abundance of *TTN* missense variants in normal cohorts (Xiao et al., 2021), their role in disease is harder to evaluate. Nevertheless, individual *TTN* missense variants have been identified as cardiomyopathy-associated (Supplementary Table S3); the following description of individual variants will be limited to the Z-disk portion of titin:

In a study by Micheu et al., NGS was used in 45 patients with HCM, and seven novel *TTN* missense variants were identified as possibly pathogenic. The two *TTN* variants (p.Ala840Ser and p. Val17Leu) were predicted to destabilize and affect the function of titin (Micheu et al., 2021). In another study by Satoh et al., 82 patients with HCM were tested, and a novel *TTN* variant (p.Arg740Leu) was mapped. This variant was shown to increase the binding affinity between titin and alpha-actinin (Satoh et al., 1999).

In a further study, two *TTN* variants (p.Ala743Val and p. Val54Met) were found in a cohort of 120 DCM patients. These variants were found to decrease the binding affinity between titin and alpha-actinin and the binding between titin and telthonin/T-cap (Itoh-Satoh et al., 2002).

In a study by Hastings et al., a three-generation family affected by DCM and LVNC was screened. They identified a *TTN* missense variant (p.Ala178Asp) as likely pathogenic. *In vitro* experiments documented protein degradation and destabilization, partial protein unfolding, and altered binding properties to the ligand telethonin (Hastings et al., 2016). A follow-on study by Jiang et al. generated a mouse model for the same *TTN* missense variant (p.Ala178Asp). Heterozygous mice had no detectable phenotype, however, the homozygous mouse model developed DCM at 3-month age and the binding partner telethonin was found to be absent from the Z-disks of these mice (Jiang et al., 2021).

In summary, truncating variants in *TTN* are now recognized as the leading genetic cause for DCM. In addition, individual *TTN* missense variants have been associated with cardiomyopathies, but due to the giant size of titin and the high abundance of missense variants in normal cohorts, it is challenging to evaluate variants identified in an individual by genetic testing. One solution proposed is a web application called TITINdb, which integrates information on titin structure, sequence, variant, and isoforms. This information, along with pre-computed predictions of the nucleotide variant’s impact on the protein, can facilitate a classification of titin variants (Laddach et al., 2017). Moreover, the emerging quantitative understanding of which exons are expressed in adult cardiac transcripts (often expressed as percentage spliced (Aufiero et al., 2018)), can help to disregard variants in exons which are not expressed in adult cardiac transcripts.

### 2.4 Muscle LIM protein (MLP)

#### 2.4.1 Structure and function of MLP

**FIGURE 5:**
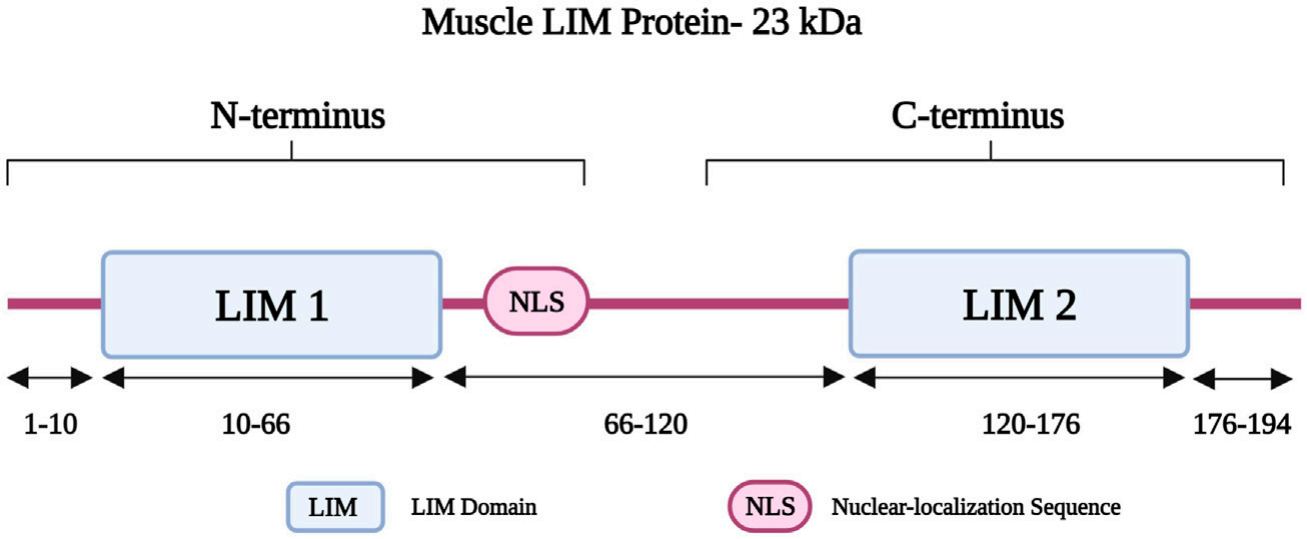
Schematic representation of the muscle LIM protein regions with different residues in the two LIM domains and the Nuclear-localization Sequence (NLS). Created with BioRender.com. Adapted from (Chauhan and Sowdhamini, 2022).

Muscle LIM protein (MLP) belongs to the cysteine-rich protein (CRP) family and is also known as cysteine- and glycine-rich protein 3 (encoded by *CSRP3*) (Geier et al., 2008). The *CSPR3* gene is located on chromosome 11p15.1 and it consists of five exons (Fung et al., 1995). MLP is 194 amino acids long and has a molecular weight of 23 kDa, consisting of two LIM domains (LIM1 and LIM2). LIM1 is followed by a nuclear-localization sequence (NLS) (Harper et al., 2000); together both form the amino-terminus of MLP (Figure 5) (Harper et al., 2000). The LIM1 domain is responsible for binding to alpha-actinin at the Z-disk (Arber et al., 1997; Harper et al., 2000). It also interacts with telethonin and calcineurin, which are involved in the detection and transduction of hypertrophic signaling pathways in response to mechanical stress (Knöll et al., 2002). MLP also has roles in calcium handling, myofibrillogenesis, and actin polymerization (Buyandelger et al., 2011). It is further important for the maintenance, assembly, and organization of the actin cytoskeleton (Arber et al., 1997; Buyandelger et al., 2011). Other roles involve mediating protein interactions and attachment of the contractile apparatus to the plasma membrane (Schallus et al., 2009).

Inactivation of *CSRP3* in mice showed significant ventricular dilation and systolic dysfunction along with substantial myocardial hypertrophy resulting in a phenotype resembling DCM (Arber et al., 1997). Since then, the *CSRP3* knockout mouse model has been used as a genetic form of heart failure in many studies (Gardiwal et al., 2007; Heineke et al., 2010; Lange et al., 2016).

#### 2.4.2 Muscle LIM protein and cardiomyopathies

Genetic variants in *CSPR3* have been linked to the pathogenesis of HCM and DCM (Supplementary Table S4). In a study by Bos et al., 389 patients with HCM were analyzed, and 12 different variants were detected in 16 HCM patients including the following *CSRP3* variants (p.Leu44Pro, p. Arg64Cys, and p. Tyr66Cys). This study also identified a frameshift variant (p. Lys42fs/165) (Bos et al., 2006). Another *CSRP3* variant (p.Arg70Trp), which is located in the T-cap binding region, was reported in an HCM patient with left ventricular hypertrophy, who had a septal wall thickness of 46 mm (Bos et al., 2006).

A study by Geier et al. screened 1100 individuals (200 HCM patients, 400 DCM patients, and 500 controls) for *CSRP3* pathogenic variants by Sanger sequencing. Three HCM-associated variants were identified (p.Leu44Pro, p. Cys58GLy, and p. Ser54Arg/p.Glu55GLy) (Geier et al., 2003). Using a yeast 2-hybrid assay, the *CSRP3* variant (p. Cys58GLy) showed impaired binding affinity to alpha-actinin (Geier et al., 2003). In another study involving 6456 HCM patients, the *CSRP3* variant (p.Cys150Tyr) was identified in 11 unrelated patients (Salazar-Mendiguchía et al., 2020). In a more recent study by Huang et al. (2022), WES was performed in a Chinese family with HCM, and the truncating *CSRP3* variant (p.Arg122Ter) was identified and considered pathogenic (Huang et al., 2022). Lipari et al. performed NGS in 29 Polish patients with HCM. The same *CSRP3* variant (p.Arg122Ter) was found in one HCM case in this cohort (Lipari et al., 2020).

To gain insights into the functional consequences of *CSRP3* variants, Ehsan et al. generated a knock-in mouse model (KI), carrying an HCM-causing variant (p.Cys58GLy). The heterozygous KI mice showed no cardiac phenotype, while the homozygous KI mice had enlarged left ventricular dimensions, decreased systolic function, and increased left ventricular mass. They further showed an 80% decrease in MLP abundance at protein level in homozygous KI mice compared to 50% in the heterozygous KI mice. In conclusion, the (p.Cys58GLy) variant could cause cardiomyopathy through protein depletion, lack of functional protein and overload of the protein degrading activity of the ubiquitin-proteasome system (Ehsan et al., 2018).

Another study showed that a *CSRP3* variant (p.Leu44Pro) can affect the structure of the MLP LIM1 domain due to the loss of hydrophobic interaction between Leucine and Phenylananine (Chauhan and Sowdhamini, 2022).

Another study by Mohapatra et al. involved 291 patients with DCM and a *CSRP3* variant (p.Lys69Arg) was identified in one patient. In this variant, the interaction between MLP and alpha-actinin two was abolished and localization of MLP was also found to be altered (Mohapatra et al., 2003). A genetic study by Hershberger involved 313 patients with DCM (divided between familial and idiopathic), and different variants were found including one *CSRP3* missense variant (p.Gly72Arg) (Hershberger et al., 2008).

For a discussion of CSRP3 p. Trp4Arg, which was initially described as a pathogenic variant, but is now considered a disease modifier, see section 4. Conclusion.

While the association between different *CSRP3* pathogenic missense variants and HCM is very well documented (Geier et al., 2003; Ehsan et al., 2018; Lipari et al., 2020), further studies are required to better understand the association between *CSRP3* variants and DCM.

### 2.5 Telethonin

#### 2.5.1 Telethonin structure and function

**FIGURE 6:**
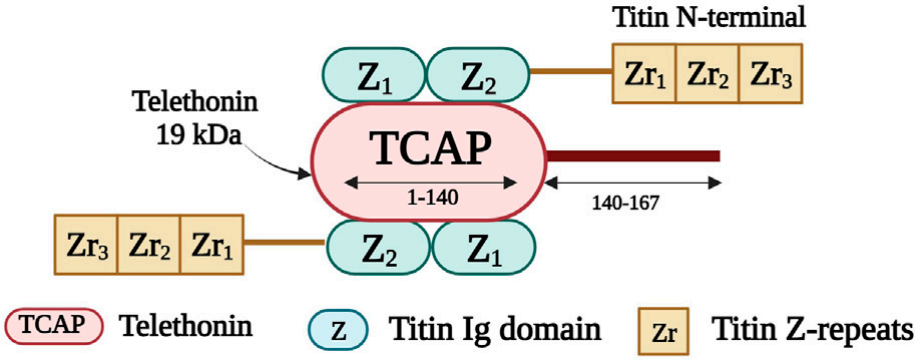
Schematic representation of the interaction taking place between the first 140 residues of telethonin and the first two titin Ig domains (Z1 and Z2). Created with BioRender.com.

Telethonin, also known as titin cap or T-cap, is a 19 kDa protein of 167 amino acids (Valle et al., 1997). It is encoded by *TCAP* on chromosome 17q12 (Anand, 2019). Telethonin is expressed in both cardiac and skeletal muscle and localizes to the Z-disk (Valle et al., 1997). It contributes to the assembly, stability, and structural integrity of the sarcomere (Anand, 2019). Telethonin is also part of a mechanosensing protein complex, together with titin, and MLP (Faulkner et al., 2001; Knöll et al., 2002). It anchors titin within the Z-disk (Gregorio et al., 1998) and it mediates the assembly of two adjacent titin molecules through the interaction of the first 140 residues of T-cap with the titin amino-terminal Ig domains Z1 and Z2 (Figure 6) (Mues et al., 1998; Lopes and Elliott, 2014). Telethonin is further implicated in T-tubule biogenesis (Al-Qusairi and Laporte, 2011); it regulates the T-tubular structure and integrity by binding to proteins in the T-tubule membrane (Ibrahim et al., 2013).

Genetic inactivation of telethonin in mice does not induce a baseline phenotype, however, heart failure response to biomechanical stress is aggravated in the mouse heart in the absence of telethonin (Knöll et al., 2011b).

#### 2.5.2 Telethonin in cardiomyopathies

Pathogenic variants in *TCAP* are rare, nevertheless they have been associated with HCM and DCM (Supplmentary Table S5). In a study by Toste et al., a novel *TCAP* variant (p.Cys57Trp) was identified in a small family with HCM presenting with left ventricular hypertrophy, atrial fibrillation, and heart failure with preserved ejection fraction (HFpEF). The variant is localized in the binding region to MLP and titin (Toste et al., 2020). In a study by Bos et al., 389 HCM patients were screened and three *TCAP* variants (p. Glu13del, p. Arg70Trp, and p. Pro90Leu) were identified in four patients (Bos et al., 2006). In another study by Hayashi et al., three *TCAP* variants were found after analyzing 346 patients with HCM (p.Thr137Ile) and 136 patients with DCM (p.Glu132GLn and p. Arg87GLn), respectively (Hayashi et al., 2004). Further qualitative tests showed that HCM-associated *TCAP* variants enhanced the interaction between telethonin and titin, while DCM-associated variants impaired the interaction between telethonin, MLP, and titin (Hayashi et al., 2004).

In summary, *TCAP* variants have been associated with both DCM and HCM, but further insights at cellular level are needed to understand the mechanism by which *TCAP* variants cause cardiomyopathies (Faulkner et al., 2001).

### 2.6 Myopalladin

#### 2.6.1 Function and structure of myopalladin

**FIGURE 7:**
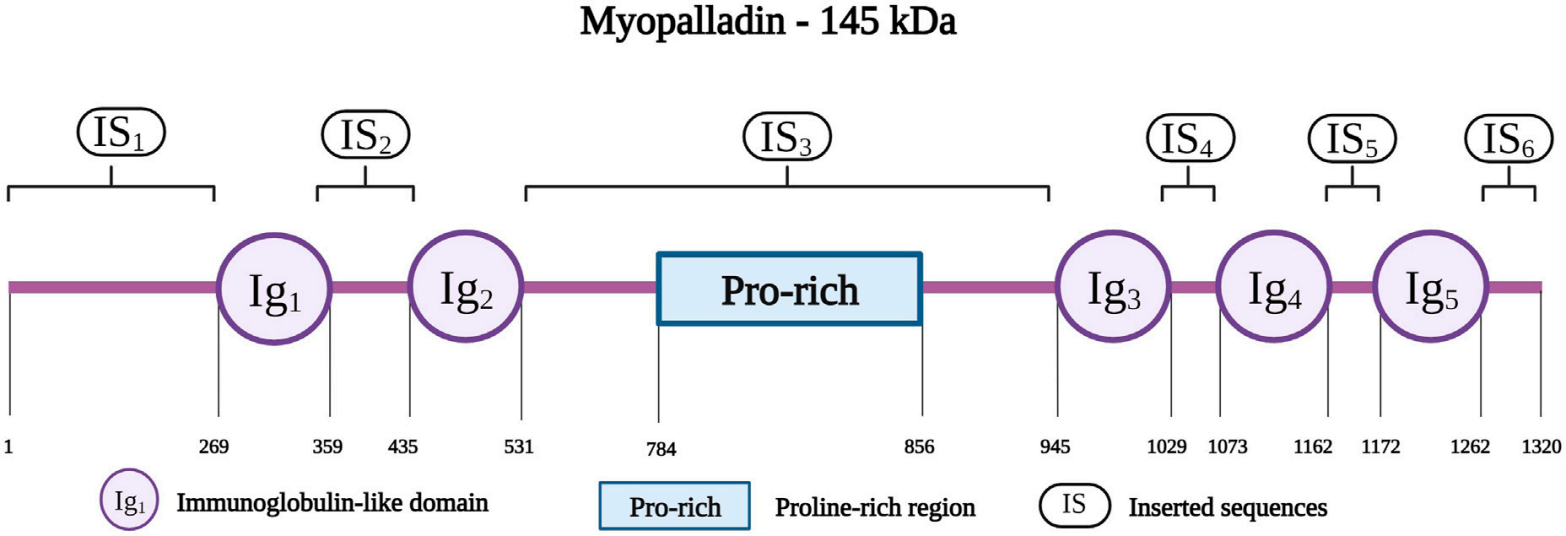
Schematic representation of the myopalladin with different residues spanning the five Ig domains (IG1 to IG5), Proline-rich region and the six inserted sequences (IS1 to IS6). Created with BioRender.com.

Myopalladin is encoded by *MYPN* on chromosome 10q21.1; the protein has a molecular weight of 145 kDa (Bang et al., 2001b). It belongs to a small family of proteins, that includes myopalladin, palladin and myotilin, which are characterized by the presence of actin-associated Ig domains (Otey et al., 2005). Myopalladin contains five Ig domains separated by six interdomain insertions (IS) where the IS3 includes a proline-rich motif (Figure 7) (Bang et al., 2001b). It is present in both the nucleus and sarcomere, with dual localization at the Z-disk and I-band (Purevjav et al., 2012).

Myopalladin interacts with several Z-disk proteins. A study showed that the myopalladin carboxy-terminal region binds to fourth and fifth Ig domains (Z4 and Z5) of titin (Filomena et al., 2021). The C-terminus also has binding sites for alpha-actinin, nebulin in skeletal muscle, and nebulette in cardiac muscle (Taylor et al., 2006). The amino-terminal domain of myopalladin binds to cardiac ankyrin repeat protein (CARP), a nuclear protein involved in hypertrophic signaling in muscles (Zou et al., 1997). Myopalladin has also been shown to bind F-actin to prevent its depolymerization (Filomena et al., 2020). In addition, myopalladin plays an important role in regulating signal transduction and gene expression during muscle stress (Purevjav et al., 2012).

In a study by Filimena et al., a myopalladin knockout mouse model showed systolic dysfunction associated with fibrosis and decreased isometric tension of the myofibrils (Filomena et al., 2021). Moreover, delays in Ca^2+^ release and uptake were observed, suggesting that altered Ca^2+^ handling is a major contributor to DCM observed in mice lacking myopalladin (Filomena et al., 2021).

#### 2.6.2 Myopalladin and cardiomyopathies

Pathogenic variants in *MYPN* have been identified and associated with DCM (Supplementary Table S6). In a study by Zhao et al., 21 patients with DCM were analyzed using NGS, and a pathogenic variant in *MYPN* (p.Glu630Lys) was identified (Zhao et al., 2015). In another study, 114 patients with DCM (65 familial and 49 sporadic) and 400 controls were included. Two heterozygous variants (p. Arg1088His and p. Ile83fsX105) were found in the familial DCM group (Duboscq-Bidot et al., 2008). In a study by Meyer et al., *MYPN* coding regions were analyzed in a cohort of 255 patients with DCM. In one DCM patients, a heterozygous missense *MYPN* variant (p.Pro961Leu) was identified and localized in the actin-binding region of myopalladin. An endomyocardial biopsy of the patient showed marked disruption of sarcomere assembly, indicating myofibrillogenesis (Meyer et al., 2013). In a genetic study by Purevjav et al., *MYPN* was examined in 484 patients with HCM and in 348 patients with DCM (Purevjav et al., 2012). Several non-sense and missense variants were found in patients with HCM (p.Arg855Ter, p. Lys153Arg, p. Ala217GLu, p. Pro841Thr, and p. Ala265Pro) and DCM (p.Ile213Val, p. Ala611Thr, and p. Ala882Thr) (Purevjav et al., 2012).

A variant initially described as pathogenic for both DCM and HCM (e.g., p. Tyr20Cys) is too common to be disease causing (MAF of 9.34 × 10^−4^), despite clear functional effects of the variant: neonatal rat cardiomyocytes expressing this variant showed disrupted intercalated discs, impaired nuclear translocation and abnormal assembly of the terminal Z-disk within the intercalated discs (Purevjav et al., 2012). However, the high MAF of this variant suggests that it is a disease modifier instead, if at all.

In conclusion, both truncating and missense variants in *MYPN* have been associated with DCM, but the role of *MYPN* variants for HCM is not fully established.

### 2.7 Nebulette

#### 2.7.1 Structure and function of nebulette

**FIGURE 8:**
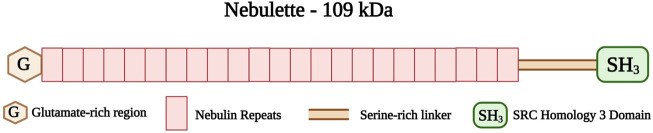
Schematic representation of the nebulette with glutamate-rich region (G) at the amino-terminus followed by 23 Nebulin repeats (35 amino acid residues each) and serine-rich linker connecting the SRC Homology 3 (SH3) domain at the carboxy-terminus. Created with BioRender.com.

Nebulette and nebulin are two thin filament-associated isoforms found in the cardiac and skeletal muscles, respectively. They belong to the nebulin family, which consists of different actin-binding proteins (Moncman and Wang, 1999). Nebulette and nebulin are encoded by the same gene, *NEBL,* located on chromosome 10p12 (Li et al., 2004). Nebulette, the cardiac-specific isoform, has a small molecular weight (109 kDa) compared to nebulin (800 kDa) (Millevoi et al., 1998). The carboxyl-terminus of nebulette is located at the Z-disk, whereas its amino-terminus protrudes outside the Z-disk along the I-band at approximately 150 nm (Bang and Chen, 2015). The nebulette structure consists of a glutamate-rich region followed by 23-residue nebulin repeats of 35 amino acid residues each (Figure 8) (Moncman and Wang, 1995; Millevoi et al., 1998). A serine-rich linker region connects the nebulin repeats to a Src homology (SH3) domain located at the carboxy-terminus of nebulette (Arimura et al., 2000; Bang and Chen, 2015).

Nebulette has a dual function, it acts as a Z-disk structural protein and further regulates signaling pathways between the nucleus and Z-disk during cardiac mechanical stretching (Ram and Blaxall, 2010). Nebulette contributes to the assembly of Z-disks (Ram and Blaxall, 2010), where it stabilizes and aligns actin filaments and cytoskeletal structures (Littlefield and Fowler, 2008). It is also involved in actin cytoskeleton organization (Ader et al., 2019). In addition, the SH3 domain of nebulette interacts with some proteins including filamin C (Holmes and Moncman, 2008), myopalladin (Bang et al., 2001b), alpha-actinin 2 (Esham et al., 2007), tropomyosin, and troponin T (Ogut et al., 2003). Through these interactions with various binding Z-disk-associated proteins, nebulette may participate in the regulation of signaling pathways (Ader et al., 2019).

A study by Mastrototaro et al. involved a knockout mouse model for nebulette. This model displayed normal cardiac function under basal conditions and in response to transaortic constriction (TAC). Molecular analysis showed no cardiac abnormalities. Nevertheless, transmission electron microscopy showed widening of the Z-disk, suggesting an essential role of nebulette in Z-disk integrity. Moreover, an upregulation in cardiac stress-responsive genes was observed, suggesting the presence of chronic cardiac stress (Mastrototaro et al., 2015).

#### 2.7.2 Nebulette and cardiomyopathies

Different pathogenic variants in the *NEBL* gene are associated with DCM and HCM (Supplementary Table S7). In a study by Perrot et al., a cohort of 217 HCM patients, 148 DCM patients, and 320 controls was investigated. Sequencing of 28 exons of *NEBL* identified three rare heterozygous missense variants in six different patients. The (p.His171Arg) variant was found in two patients with HCM. Two other variants (p.Gln581Arg and p. Ser747Leu) were detected in three patients with DCM (Perrot et al., 2016).

In a study by Purevjav et al., a pathogenic variant (p.Gln128Arg) was identified and mapped to nebulette ABD. A transgenic mouse model with the (p.Gln128Arg) variant was embryonic lethal. Embryos harvested up to E12.5 showed an irregular localization pattern of nebulette with possible dissociation from the Z-disk. In addition, there was an impairment in the expression of the desmin protein (Purevjav et al., 2010).

A genetic study screened several genes in six cohorts of patients diagnosed with LVNC. Four *NEBL* missense variants (p.Ala726Thr, p. Ser863Cys, p. Ser296GLy, and p. Asn330Ser) were found in four cases out of a total of 278 patients (Mazzarotto et al., 2021).

In conclusion, *NEBL* variants have been described as rare causes of HCM, DCM and LVNC. However, many *NEBL* variants initially described do not meet stringent MAF cut-off criteria (Supplementary Table S7), and cannot be considered pathogenic, despite showing functional abnormalities in transgenic mouse studies (Purevjav et al., 2010; Maiellaro-Rafferty et al., 2013).

### 2.8 Nexilin

#### 2.8.1 Nexilin structure and function

**FIGURE 9:**
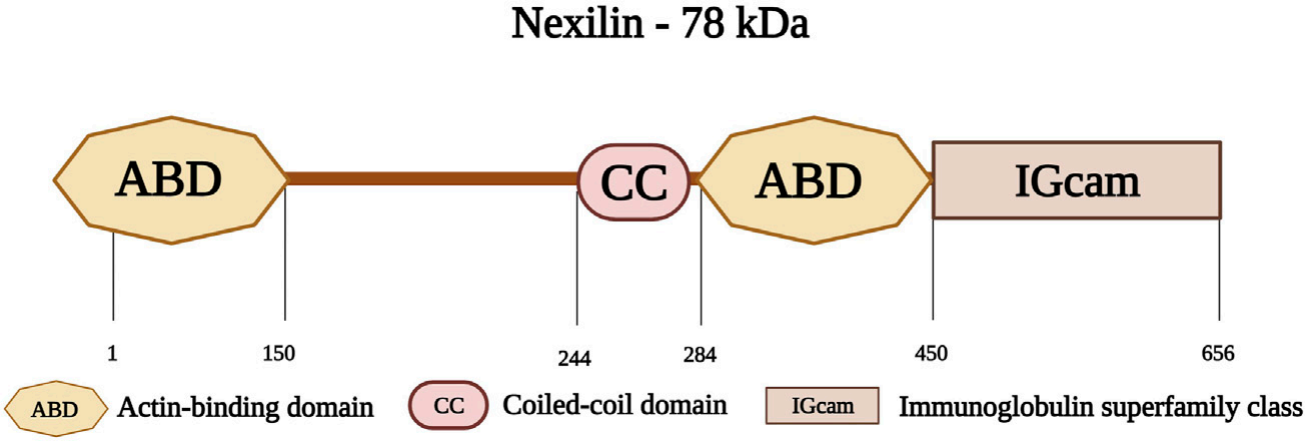
Schematic representation of nexilin with its first actin-binding domain (ABD) at the amino-terminus followed by a coiled-coil domain (CC) at the middle region and a second ABD. The carboxy-terminus consists of an Immunoglobulin superfamily class domain (IGcam). Created with BioRender.com.

Nexilin is an F-actin-binding protein, encoded by the gene *NEXN* on chromosome 1p31.1. Nexilin has a molecular weight of 78 kDa and consists of 656 amino acids (Ohtsuka et al., 1998). Its amino-terminal consists of an ABD, followed by a coiled-coil (CC) domain. This is followed by another ABD, and a carboxy-terminal immunoglobulin superfamily class (IGcam) domain (Figure 9) (Ohtsuka et al., 1998; Hassel et al., 2009). Nexilin is located at the sarcomeric Z-disk and interacts with highly specialized sarcoplasmic reticulum (SR) proteins, termed junctional membrane complexes (Liu et al., 2019). It is considered an essential component of this complex where it participates in the development and maintenance of cardiac T-tubules (Liu et al., 2020; Spinozzi et al., 2020). In addition, this protein is essential for cardiac function and development (Johansson et al., 2022), and is involved in cell adhesion and migration in various tissues including cardiac and skeletal muscle tissues (Wang et al., 2005). Other roles include maintaining the integrity of the Z-disks against the tension generated within the sarcomere (Lopes and Elliott, 2014).

In a study by Spinozzi et al., a nexilin knockout mouse model was generated. The authors studied isolated cardiomyocytes from the mouse model. Nexilin loss was associated with reduced cardiac function and progressive DCM, characterized by increased left ventricular diameter, ventricular wall thinning, and reduced ejection fraction. Other results revealed disorganization in the T-tubule network, with a significant decrease in the transversal component, indicating the importance of nexilin in the maintenance and organization of the transverse-axial tubule system in adult cardiomyocytes (Spinozzi et al., 2020).

Another study by Aherrahrou et al., involved a nexilin KO mouse model. The heart weight of the knockout mice was 2.3-fold higher than the WT hearts and knockout mice developed rapid progressive cardiomyopathy on day 6, had decreased systolic cardiac function, left ventricular dilation, and wall thinning. In addition, elastin and collagen deposits were observed within the cavity of the left ventricle, resembling features of endomyocardial fibroelastosis (Aherrahrou et al., 2016).

#### 2.8.2 Nexilin and cardiomyopathy

Several studies have shown that pathogenic variants *NEXN* can lead to progressive DCM (Spinozzi et al., 2020; Bruyndonckx et al., 2021). A study by Johansson et al. studied a case of a female with three consecutive pregnancies and intrauterine fetal deaths caused by a lethal form of DCM. WES of the fetus revealed homozygosity for the *NEXN* variant (p.Ile435SerfsTer3). Histology revealed cardiomegaly and endocardial fibroelastosis, with immunohistochemical staining using a polyclonal nexilin antibody showing loss of striation in the heart (Johansson et al., 2022).

In a study by Hass et al., NGS was used in 639 patients with DCM. Five missense *NEXN* variants (p.Glu110GLn, p. Gly157Val, p. Thr363Arg, p. Glu485Lys, and p. Thr666Ala) were identified in the DCM cohort. One non-sense variant (p.Arg392Ter) and one deletion (p.Glu468del) were also identified (Haas et al., 2015).

Another study by Wang et al. involved 121 patients with HCM and a missense variant (p.Gln131GLu) was identified in two patients. This variant was identified in exons five of the *NEXN* gene and was absent in 384 controls. Cellular transfection studies showed that the variant affecting conserved amino acid residues, led to local accumulation of nexilin. Moreover, the expressed fragment of the nexilin actin-binding domain with the (p.Gln131GLu) variant could not bind F-actin or alpha-actinin (Wang et al., 2010).

Another study examined a 11-years-old asymptomatic boy with DCM. Genetic testing revealed a heterozygous *NEXN* variant (p.Gly650del) inherited from his father, who was clinically characterized by mild DCM (Bruyndonckx et al., 2021). This study involved another patient who presented with left ventricular systolic dysfunction, and she required mechanical ventilation and continuous inotropic support. A few weeks later, the patient died once her therapy was discontinued. A microscopic examination of the heart revealed endomyocardial fibroelastosis. WES revealed a homozygous (p.Arg392Ter) variant. This variant was mapped to the actin-binding domain of nexilin, resulting in a premature stop codon, and non-functional nexilin protein (Bruyndonckx et al., 2021).

In a further study by Hassel et al., 1,000 patients with DCM were screened and three *NEXN* variants (p.Gly650del, p. Tyr652Cys, and p. Pro611Thr) were identified in nine individuals. These variants were mapped to the nexilin carboxy-terminal Ig domain. In patients with these *NEXN* variants, a disruption of sarcomeric units and detached Z-disks were observed. A zebrafish model was used to demonstrate the effects of these variants on atrial and ventricular chambers. Electron microscopy was performed on the zebrafish embryonic hearts having the *NEXN* variants (p.Tyr652Cys and p. Pro611Thr) and the results showed a disruption in the Z-disk integrity and architecture. Further, the zebrafish model was also subjected to a three bp deletion in nexilin gene (1948-1950del), leading to a loss of glycine at position 650 (p.Gly650del). Embryonic hearts of zebrafish carrying the (p.Gly650del) variant were severely dilated, with a marked reduction in systolic function (Hassel et al., 2009).

In a study by Liu et al., CRISPR/CAS9 technology was used to generate a mouse model for the same in frame deletion (p.Gly650del). Homozygous mice with the (p.Gly650del) variant exhibited a progressive DCM phenotype characterized by disorganization of the transverse-axial tubular system and a reduction in T-tubule formation, underpinning the importance of the functional *NEXN* gene in cardiac function and tubular system organization (Liu et al., 2020).

Therefore, the *NEXN* variants described (Supplementary Table S8) contribute to the further understanding of the importance of nexilin protein in cardiac contractility and function. These studies add to the current knowledge of the role of nexilin in the Z-disk and its pertubations in DCM.

## 3 Z-disk associated proteins

### 3.1 Desmin

#### 3.1.1 Desmin structure and function

Desmin, encoded by *DES* on chromosome 2q35, belongs to the intermediate filament family and is found mainly in the cardiac and skeletal muscles (Hnia et al., 2015). In this review, we will be discussing desmin as a protein associated with the Z-disk. Desmin extends from the Z-disks of the superficial myofibrils to the Z-region of the costameres in the sarcolemma (Capetanaki et al., 2015). It links the contractile apparatus to the costameres of the plasma membrane (Hayman et al., 2000; Clemen et al., 2013; Capetanaki et al., 2015).

**FIGURE 10:**
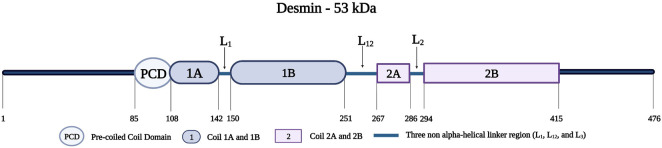
Schematic representation of the desmin protein consisting of a pre-coiled coil domain, followed by four coiled domains (Coil 1A, 1B, 2A, and 2B) that are separated by three linker regions (L1, L12, and L2). Created with BioRender.com. Adapted from (Bär et al., 2005).

Desmin is a 53-kDa protein; it assembles as oligomers to form filaments (Vicart et al., 1996; Clemen et al., 2013). Desmin monomers, consisting of 470 amino acids, have a tripartite structure with an amino-terminal head, an amphipathic central alpha-helical rod domain consisting of a pre-coiled coil domain (PCD) followed by four segments (Coil 1A, 1B, 2A, and 2B), three short non-helical rod segments (L1, L12, and L2), and a tail domain (Figure 10) (Goldfarb and Dalakas, 2009; Capetanaki et al., 2015). The amino-terminal head is important for the proper assembly of mature desmin monomers (Brodehl et al., 2018). This assembly occurs when two desmin monomers form a dimer and two desmin dimers form a tetramer that aligns in an antiparallel fashion with two coils overlapping (Coil 1A and 1B) (Clemen et al., 2013). Eight tetramers polymerize and assemble longitudinally to form a desmin intermediate filament (Clemen et al., 2013; Capetanaki et al., 2015).

Furthermore, desmin is an important cytoarchitectural protein that plays several crucial roles in maintaining the structure of myocytes and their organelles. For instance, desmin links the contractile apparatus to the cytoskeleton of the sarcolemma and various cytoplasmic organelles, including the nucleus, mitochondria, and lysosomes (Milner et al., 1999; Capetanaki et al., 2015; Dayal et al., 2020). In addition, desmin facilitates transport and mechanochemical signaling between different cell organelles and the extracellular matrix (Capetanaki et al., 2015). Desmin also plays a vital role in the maintenance of muscle contraction (Singh et al., 2020) and transduction of longitudinal stretch signals (Frank et al., 2007). Other roles include maintaining the structural and functional integrity of the cardiomyocyte structure and organizing the cytoskeleton (Goldfarb and Dalakas, 2009; Dayal et al., 2020). Furthermore, desmin interacts with mitochondria to ensure their proximity to the A- and I-bands. This interaction ensures that ATP produced in mitochondria can meet the energy requirements of the contractile apparatus through close proximity (McLendon and Robbins, 2011). A study further studied the interaction between desmin and the mitochondrial voltage-dependent anion channel (VDAC). This channel has several functions including the modulation of ATP transportation to the outer mitochondrial membrane, and controlling the transport of metabolites between the mitochondria and cytoplasm. The study used a desminopathy rat model and showed an increased accumulation of VDAC1 in areas of muscle fibers that highly stained for desmin (Li et al., 2016).

In a study by Elsnicova et al., a desmin knock-out mice model was used to assess for metabolic myocardial phenotyping. The analysis revealed a decrease in mitochondrial number, mitochondrial defects, and impaired fatty acid transport, activation and catabolism (Elsnicova et al., 2022). Another study used a desmin knock-out mouse model and demonstrated cardiomyocytes death leading to calcification and fibrosis (Milner et al., 1999).

#### 3.1.2 Desmin and cardiomyopathies

Genetic variants in *DES* can lead to dysregulation of mechanochemical signaling processes in the cardiac contractile apparatus, resulting in different cardiomyopathies (Supplementary Table S9). A study by Harada et al. identified a novel homozygous *DES* variant (p.Thr219Pro) in a 20-year-old patient with HCM. The patient’s parents were heterozygous carriers of this variant without a cardiac phenotype, suggesting recessive inheritance. This variant was mapped to the 1B α-helix domain of desmin and was determined to be disease-causing for HCM (Harada et al., 2018). Another study reported a case of an 8-year-old girl with HCM and congenital atrioventricular heart block. A *de novo* heterozygous *DES* missense variant (p.Arg406Trp) was suggested to be disease-causing (Oka et al., 2021).

In a study by Miyamoto et al., 265 Japanese patients with DCM were examined, and a *DES* missense variant (p.Ile451Met) in exon eight was identified in three patients (Miyamoto et al., 2001). The same variant was studied by Li et al. in 44 patients with DCM and 460 controls. This *DES* variant was identified in four DCM patients and was absent in the control group. It localizes to the carboxy-terminus (Li et al., 1999). A study by Mavroidis et al. generated a mouse model expressing the same *DES* variant (p.Ile451Met) and showed that mutant desmin had architecture defect and an altered desmin Z-disk localization (Mavroidis et al., 2008).

In a study by Fischer et al., a large German family showing ECG abnormalities was tested using both Sanger sequencing and NGS. Two novel *DES* variants (p.Ile402Thr and p. Glu410Lys) were identified. Both localized to the second coil region (Coil 2B) of the rod domain. Individuals carrying these variants have DCM or ACM. Cardiomyocytes transfected with the two variants showed defects in filament assembly, resulting in desmin aggregates. The two variants were classified as disease-causing (Fischer et al., 2021).

In another study, RCM was investigated by screening for *DES* variants in four families (with a total of 19 individuals). Three *DES* variants (p.Arg16Cys, p. Thr453Ile, and p. Arg406Trp) were found in nine patients (Arbustini et al., 2006).

Another study by Herrmann et al. reported a case of an adolescent patient with RCM. A heterozygous *DES* variant (p.Arg406Trp) was identified by NGS. A knock-in mouse model for this variant showed aggregates of desmin and the absence of desmin filaments at the level of the intercalated discs on microscopic analysis. Other findings were structural changes within the intercalated discs, as demonstrated by the abnormal organization of desmoplakin, plectin, and connexin. In addition, cell transfection studies suggested that desmin formed unusually thick filaments organized into complex filament aggregates and fibrillar sheets. Hence, the study concludes that the *DES* variant (p.Arg406Trp) can cause cardiomyopathy with severe disarrangement of intercalated disks (Herrmann et al., 2020).

A study by Klauke et al. screened 22 patients with ACM and several *DES* variants were detected (see Supplementary Table S9). The *DES* variant (p.Asn116Ser) was found in one ACM patient with heart failure requiring transplantation. This variant was located to segment 1A of desmin rod domain. Immunohistochemical studies of the myocardial sections showed desmin and myotilin accumulation of immunoreactive aggresomes in both the right and left ventricle (Klauke et al., 2010). Another study by Brodehl et al. screened two families with different cardiac phenotypes including ACM, DCM, and atrioventricular block. Two novel *DES* variants were identified (p.Ala120Asp and p. His326Arg) and followed by *in vitro* experiments. The cellular model with the (p.Ala120Asp) variant showed severe intrinsic defect in filament formation causing cytoplasmic aggregates of desmin. *Ex vivo* analysis also showed severe cytoplasmic aggregate formation and a loss of desmin staining within the intercalated disk (Brodehl et al., 2013).

Furthermore, some studies have addressed desmin-related cardiomyopathy (DRM), defined as a rare genetic cardiac and skeletal muscle disease caused by *DES* variants. This disease is characterized by the combination of skeletal muscle weakness and DCM. A case report of a Japanese female with a dilated left ventricle and reduced left ventricular wall motion was studied. A disease-causing *de novo* missense *DES* variant (p.Arg454Trp) was identified (Tamiya et al., 2020). Another study by Olivé et al. included ten patients with DRM and 123 healthy controls. The *DES* pathogenic variant (p.Arg406Trp) was identified in two patients (Olivé et al., 2004). Another study targeted DRM by using a transgenic mouse model with a 7-amino-acid deletion (p.Arg173_Glu179del). Mice heterozygous for the variant exhibited myofibrillar disarray and left ventricular hypertrophy. Other findings included electron-dense granular and filamentous desmin aggregates located in the perinuclear region of cardiomyocytes and the intermyofibrillar space (Wang et al., 2001).

In conclusion, *DES* pathogenic variants are highly associated with different types of cardiomyopathies. The association of *DES* with DCM is the best documented (Li et al., 1999; Miyamoto et al., 2001), in addition to variants affecteing both the heart and skeletal muscle in DRM.

### 3.2 Obscurin

#### 3.2.1 Structure and function of obscurin

**FIGURE 11:**
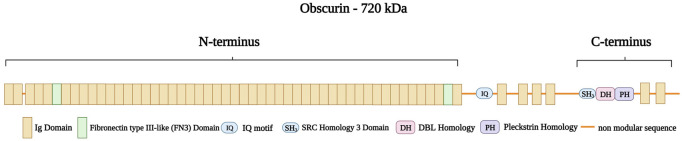
Schematic representation of obscurin A with the amino-terminus consisting of 49 Ig domains (brown) with two Fibronectin type III-like (FN3 domain, green) spanning the boundaries of the amino-terminus. This structure is followed by a non-modular sequence intersected by an IQ motif and four Ig domains. The caboxy-terminus consists of a SRC Homology three Domain (SH3), a DBL Homology domain (DH), and a Pleckstrin Homology domain (PH) followed by two Ig domains. Created with BioRender.com.

Obscurin is encoded by *OBSCN* in the chromosomal region 1q42.13 (Russell et al., 2002). The protein ranges in size from 40 to 870 kDa (Young et al., 2001) and has multiple isoforms: Obscurin-A (720 kDa), Obscurin-B (870 kDa), intermediate Obscurin (260–600 kDa), and small Obscurin (40–260 kDa) (Manring et al., 2017). Obscurin A and B are abundantly expressed in both skeletal and cardiac muscle, while the smaller obscurins are located in the nucleus (Bowman et al., 2007). Obscurin consists of 88-amino acid Ig-like repeats (Nagase et al., 2000) with an amino-terminus consisting of 49 Ig domains and two fibronectin type III-like (FN3) domains (Young et al., 2001). An IQ motif and four additional Ig domains follow this region. The carboxy-terminus of obscurin contains an SH3 domain, followed by a tandem dbl homology (DH), a pleckstrin homology (PH) domain, and two further Ig repeats (Figure 11) (Young et al., 2001; Russell et al., 2002; Kontrogianni-Konstantopoulos et al., 2006). Furthermore, obscurin A and B share the same modular architecture but differ in their carboxy-termini with obscurin-A having an ankyrin binding site and obscurin-B having two active Ser/Thr kinase domains belonging to the myosin light chain kinase family (Russell et al., 2002)

Obscurin localizes to both the Z-disk as well as to M-bands (Young et al., 2001). The obscurin Ig domains (48–49) localize to the Z-disk where they interact directly with the peripheral Z-disk Ig domains (Z9 and Z10) of titin during early development and myofibrillogenesis (Young et al., 2001). During the progression of myofibrillogenesis, obscurin is detectable at the M-band (Bang et al., 2001a; Young et al., 2001) and is implicated in the regulation of M-band assembly and structure (Benian et al., 1996). In another study, obscurin domains were found to target to the Z-disk region and were shown to interact with the titin Novex-3 isoform (Bang et al., 2001a). Obscurin also interacts with calmodulin in a calcium-dependent manner through its Ig_52_ domains (Young et al., 2001). It further binds to small ankyrin 1, an integral component of the sarcoplasmic reticulum (SR) membrane (Kontrogianni-Konstantopoulos et al., 2006).

Obscurin has multiple functions in facilitating myofibrillogenesis and contributing to sarcomeric organization and stabilization (Randazzo et al., 2017). Obscurin maintains sarcomere integrity and aligns the SR with the contractile apparatus (Subramaniam et al., 2022). Other roles include the maintenance and assembly of A- and M-bands (Kontrogianni-Konstantopoulos and Bloch, 2005) to transmit contractile forces across the sarcolemma (Subramaniam et al., 2022).

A study by Blondelle et al. showed that the global knockout of obscurin is embryonically lethal (Blondelle et al., 2019). Other studies have investigated the consequence of lack of obscurin on the sarcroplasmic reticulum (SR) (Lange et al., 2009; Randazzo et al., 2013). The lack of obscurin in skeletal muscles has been linked to changes in longitudinal SR architecture and sarcolemmal integrity (Lange et al., 2009; Randazzo et al., 2013). Another study by Grogan et al. used a mice model with a deletion in Ig58/59 of obsucrin. These 58 and 59 Ig domains are essential to obscurin as they mediate binding to titin. The mice model developed left ventricular hypertrophy, contractile impairment, atrial enlargement, and arrhythmia (Grogan et al., 2020).

#### 3.2.2 Obscurin and cardiomyopathy

Several variants in *OBSCN* have been associated with different cardiomyopathies (Supplementary Table S10). A study by Arimura et al. included 144 Japanese patients with HCM and 288 healthy controls. Two novel *OBSCN* variants (p.Arg4344GLn and p. Ala4484Thr) were identified. Functional studies revealed that (p.Arg4344GLn) impairs the ability of obscurin to bind to the Z9-Z10 titin domains, thereby impairing Z-disk localization of obscurin (Arimura et al., 2007). In another study by Hu et al., a knock-in mouse model with the same *OBSCN* variant (p.Arg4344GLn) was investigated, but did not reveal any cardiac pathology (Hu and Kontrogianni-Konstantopoulos, 2020). In contrast, a study by Fukuzawa et al. used a different knock-in mouse model for the same variant (p.Arg4344GLn) and described a cardiomyopathy-like phenotype with abnormal Ca^2+^handling. However, no changes in the thermostability or binding of obscurin to Z9-Z10 titin were detected (Fukuzawa et al., 2021). A further knock-in mouse model for this variant (p.Arg4344GLn) showed that the expression patterns of the sarcomeric and Ca^2+^ cycling proteins were unchanged. However, isolated cardiomyocytes from a 1-year-old knock-in hearts exhibit an increase in Ca^2+^ transients in the SR, as well as faster contraction kinetics (Hu et al., 2017).

In a study by Marston et al., WES was performed on 30 patients with DCM, and five potentially disease-causing *OBSCN* variants (p.Glu963Lys, p. Val2161Asp, p. Phe2809Val, p. Asp5966Asn, and p. Arg4856His) were identified in four patients (Marston et al., 2015). Rowland et al. studied a cohort of 325 DCM and 10 LVNC patients. Two *OBSCN* variants (p.Thr7266ArgfsTer53 and p. Ser7947ProfsTer82) were identified in two LVNC patients and one *OBSCN* variant (p.Ala7950ProfsTer79) was identified in one DCM patient (Rowland et al., 2016). In a study by Xu et al., a cohort of 74 patients with HCM was studied. WES was performed and two *OBSCN* missense variants (p.Arg5215His, and p. Gly7500Arg) and four frameshift variants (p. Ala996fs, p. Ala1088fs, p. Ala1272fs, and p. Ala1640fs) were identified (Xu et al., 2015).

A further study by Chen et al. generated iPSC-CMs from a 46-year-old female diagnosed with ACM, carrying a frameshift variant in *OBSCN* (p.Leu5218fs). These cells showed impaired localization and decreased expression of the obscurin anchoring protein (Ank1.5), supporting a pathogenic role of this variant (Chen et al., 2020).

In conclusion, most of the documented *OBSCN* variants are associated with HCM and DCM. However, the effect of these variants on Z-disk assembly and cardiac contractility have not been well elucidated.

## 4 Conclusion

As discussed in the review, Z-disk proteins and Z-disk-associated proteins play a critical role in maintaining the structure and function of the cardiac contractile apparatus. The interaction of these different proteins within the Z-disk allows the structure to perform its role as a signaling hub where it transmits mechanical and biochemical signals (Frank and Frey, 2011; Filomena et al., 2021). A large number of pathogenic variants of the Z-disk proteins have been shown to associate with several types of cardiomyopathies. These genetic variants lead to disruption or inactivation of the corresponding proteins thus advancing research on rare cardiomyopathies by providing a rich source of information on disease phenotypes (Karczewski et al., 2020).

Over the past decade, the advancement and wide application of high-throughput sequencing technologies have made genetic analyses more accessible and accurate (Karczewski et al., 2020). In addition, sequencing of normal cohorts has revealed insight into the surprisingly large genetic variation not associated with disease (GnomAD, 2014). Hence, minor allelic frequency (MAF) cut-offs have become more stringent for monogenetic disease, with a MAF threshold of less than 1 × 10^−4^ for autosomal dominant cardiomyopathies (Walsh et al., 2017). This will facilitate the detection of disease-causing variants and will allow earlier disease diagnosis and timely treatment.

Our increasing knowledge about genetic variant in diseased and normal cohorts as well as biological functions of proteins also means that not all genetic variants historically reported would still be considered pathogenic today. This is also reflected in a more nuanced classification of the likelihood of variants to be pathogenic (Richards et al., 2015).

Furthermore, an important focus in genetic studies is the distinction between monogenic and polygenic risk variants. Monogenic risk variants are defined as a single variant that disrupt a physiological pathway with a major impact on the disease phenotype (Fahed et al., 2020). In contrast, polygenic risk includes multiple variants with small effect in different pathways (Fahed et al., 2020). Recent studies have shown that combinations of common variants with small effect sizes can cause HCM and DCM that is phenotypically similar to monogenic disease in the affected individual (Harper et al., 2021; Tadros et al., 2021). It is important to analyze both monogenic risk and polygenic risk variants (Walsh et al., 2017), however, very few studies have examined the interplay between them.

Another important focus is to differentiate between disease-causing and disease-modifying variants. A disease modifier is defined as a newly-identified variant of unknown significance that can modify the disease phenotypic outcome (Nollet et al., 2020). Such disease modifying variants may show clear functional changes to protein characteristics (e.g., stability or ligand binding), but have high prevalence in normal cohorts which contradicts them as the sole genetic cause for disease, as it would imply an implausible low penetrance (Ye et al., 2019).

A prime example is a variant in *NEBL* (p.Gly202Arg) which was initially described as disease-causing based on the phenotype of a mouse model (Purevjav et al., 2010), however it has a high MAF value (2.3 × 10^−3^), which is too high to be considered disease-causing.

Another example is CSRP3 variant (p.Trp4Arg). It was identified by Knöll et al. in a study involving 1400 individuals divided into control and DCM patients. The CSRP3 missense variant (p.Trp4Arg) impair binding to telethonin, leading to Z-disk misalignment, and eventually DCM (Knöll et al., 2002).

A study by Riaz et al. generated cellular models (iPSC-CMs and engineered heart tissues (EHTs) with the same (p.Trp4Arg) variant. The derived cardiomyocytes showed a higher decay rate in the muscle LIM protein expression, and the derived EHTs showed impaired relaxation and prolonged force generation (Riaz et al., 2022). Despite this variant showing clear phenotypic changes in clinical studies, iPSC-CM cultures and engineered heart tissues; it has an implausible high MAF value (2.28 × 10^−3^) and now considered a disease modifier (Knöll et al., 2010).

Moreover, genetic variants may predispose different responses to environmental factors. It is emerging that genetic variants in *TTN* are enriched in cohorts of cardiac disease caused by pregnancy, alcohol abuse or cancer therapy (Ware et al., 2016; Ware et al., 2018; Garcia-Pavia et al., 2019), suggesting that an aggravated response to an environmental trigger could be caused by the presence of the *TTN* variant.

Finally, the field of genetic analysis field is very broad, and new bioinformatics and experimental approaches are needed to facilitate analysis of the pathogenicity of different variants in the future. The continued development and improvement of iPSC-CMs is an exciting trend for disease modelling [reviewed in (Reilly et al., 2022)], also of Z-disk associated cardiomyopathies, and will complement whole organ investigations in animal models [reviewed in (Sheikh and Chen, 2007)] to allow better insights into disease mechanisms.
