# Supplementary material for: Structural and signaling proteins in the Z-disk and their role in cardiomyopathies
Source: Front Physiol. 2023 Mar 2;14:1143858. doi: 10.3389/fphys.2023.1143858 (PMC10017460; doi:10.3389/fphys.2023.1143858)
Supplement: Supplementary file 1 [file DataSheet1.docx]

Supplementary Material

Structural and Signaling Proteins in the Z-disk and their Role in Cardiomyopathies

Maya Noureddine^1*^, Katja Gehmlich^1,2*^

^1^Institute of Cardiovascular Sciences, College of Medical and Dental Sciences, University of Birmingham, Birmingham, United Kingdom

^2^Cardiovascular Medicine, Radcliffe Department of Medicine and British Heart Foundation Centre of Research Excellence Oxford, University of Oxford, Oxford, United Kingdom

*** Correspondence:**Maya Noureddine
mmn207@student.bham.ac.uk

Katja Gehmlich
k.gehmlich@bham.ac.uk

# Supplementary Tables

The below supplementary tables summarize **missense variants** in Z-disk and Z-disk associated proteins. They show the amino acid and nucleotide changes as well as the associated cardiomyopathies. Data was retrieved from the QIAGEN Human Gene Mutation Database (HGMD, accessed 12/2022). The Minor Allelic Frequency (MAF) of each variant was retrieved from GnomAD (Genome Aggregation Database, accessed 12/2022). Variants with a MAF higher than > 1 x 10^−4^ are marked in Italics with *.

**Supplementary Table 1: *ACTN2* missense variants reported in different types of cardiomyopathies**

Abbreviations: DCM, dilated cardiomyopathy; HCM, hypertrophic cardiomyopathy; LVNC, left ventricular noncompaction; *variants with a Minor Allele Frequency (MAF) > 1 x 10^−4^. MAF retrieved from the Genome Aggregation Database (gnomAD).

| **Alpha-actinin 2 Missense Variants** | **Nucleotide Change** | **Disease-associated** | **MAF on GnomAD** | **Pubmed**  **ID** | **Reference** |
| --- | --- | --- | --- | --- | --- |
| **Gln9Arg* | *c.26A>G* | *DCM* | *7.22 x 10^-4^* | *14567970* | *Mohapatra (2003) Mol Genet Metab 80:207* |
| Met20Val | c.58A>G | HCM | Absent | 27532257 | Walsh (2017) Genet Med 19:192 |
| Phe44Leu | c.130T>C | DCM | Absent | 27532257 | Walsh (2017) Genet Med 19:192 |
| Pro85Ser | c.253C>T | HCM | Absent | 31513939 | Robyns (2020) Eur J Med Genet 63 |
| Met92Val | c.274A>G | HCM | Absent | 27532257 | Walsh (2017) Genet Med 19:192 |
| Arg93Gln | c.278G>A | HCM | 2.83 x 10^-5^ | 31737537 | Marschall (2019) Cardiovasc Diagn Ther 9:S292 |
| Gly111Val | c.332G>T | HCM | Absent | 17097056 | Theis (2006) Biochem Biophys Res Commun 351:896 |
| Val115Met | c.343G>A | DCM | Absent | 20474083 | Zimmerman (2010) Genet Med 12:268 |
| Ala119Thr | c.355G>A | HCM | Absent | 20022194 | Chiu (2010) J Am Coll Cardiol 55:1127 |
| Ser147Leu | c.440C>T | HCM | 3.98 x 10^-6^ | 32492895 | Kim (2020) J Clin Med 9: |
| Asn175Tyr | c.523A>T | RCM | Absent | 27662471 | Kostareva (2016) PLoS One 11:e0163362 |
| Leu186Phe | c.556C>T | DCM | 6.36 x 10^-5^ | 27532257 | Walsh (2017) Genet Med 19:192 |
| Met216Val | c.646A>G | DCM | 2.39 x 10^-5^ | 31983221 | Mazzarotto (2020) Circulation 141:387 |
| Met228Thr | c.683T>C | HCM | Absent | 25173926 | Girolami (2014) Circ Cardiovasc Genet 7:741 |
| **Asp230Glu* | *c.690T>A* | *HCM* | *1.13 x 10^-4^* | *27532257* | *Walsh (2017) Genet Med 19:192* |
| Met246Val | c.736A>G | DCM | Absent | 27532257 | Walsh (2017) Genet Med 19:192 |
| Thr247Met | c.740C>T | HCM | Absent | 31680489 | Prondzynski (2019) EMBO Mol Med 11:e11115 |
| **Arg298His* | *c.893G>A* | *LVNC* | *4.46 x 10^-4^* | *28798025* | *Miszalski-Jamka (2017) Circ Cardiovasc Genet 10:e001763* |
| Arg307Gln | c.920G>A | DCM | 1.41 x 10^-5^ | 31983221 | Mazzarotto (2020) Circulation 141:387 |
| Leu320Arg | c.959T>G | DCM | 7.95 x 10^-6^ | 30630173 | Fan (2019) Cytogenet Genome Res 157:148 |
| Asp325Tyr | c.973G>T | HCM | Absent | 33297573 | Micheu (2020) Diagnostics (Basel) 10:1061 |
| Arg327Cys | c.979C>T | HCM | 1.99 x 10^-5^ | 27532257 | Walsh (2017) Genet Med 19:192 |
| Arg328Gln | c.983G>A | HCM | 1.41 x 10^-5^ | 28790153 | Burns (2017) Circ Cardiovasc Genet 10:e001666 |
| Pro332Gln | c.995C>A | DCM | 3.98 x 10^-6^ | 31983221 | Mazzarotto (2020) Circulation 141:387 |
| Lys334Asn | c.1002G>C | LVNC | Absent | 26688388 | Chanavat (2016) Clin Chim Acta 453:80 |
| **Thr347Met* | *c.1040C>T* | *HCM* | *1.34 x 10^-4^* | *33658040* | *Chung (2021) J Cardiovasc Magn Reson 23:18* |
| Gln349Leu | c.1046A>T | DCM | 1.06 x 10^-5^ | 24082139 | Gonzalez-Garay (2013) Proc Natl Acad Sci U S A 110:16957 |
| Arg353Trp | c.1057C>T | HCM | 1.59 x 10^-5^ | 27532257 | Walsh (2017) Genet Med 19:192 |
| Glu387Gln | c.1159G>C | HCM | Absent | 30847666 | Lint (2019) Neth Heart J 27:304 |
| Arg398His | c.1193G>A | HCM | 1.19 x 10^-5^ | 28790153 | Burns (2017) Circ Cardiovasc Genet 10:e001666 |
| Ala432Val | c.1295C>T | DCM | 4.25 x 10^-5^ | 31983221 | Mazzarotto (2020) Circulation 141:387 |
| Thr435Thr | c.1305A>C | DCM | Absent | 32746448 | Burstein (2021) Pediatr Res 89:1470 |
| Glu436Ala | c.1307A>C | DCM | 1.59 x 10^-5^ | 31983221 | Mazzarotto (2020) Circulation 141:387 |
| Glu448Ala | c.1343A>C | HCM | Absent | 33658040 | Chung (2021) J Cardiovasc Magn Reson 23:18 |
| Glu448Lys | c.1342G>A | HCM | 3.98 x 10^-6^ | 30165862 | Lu (2018) J Transl Med 16:241 |
| Arg457Cys | c.1369C>T | HCM | 7.97 x 10^-6^ | 28790153 | Burns (2017) Circ Cardiovasc Genet 10:e001666 |
| Gln460Arg | c.1379A>G | HCM | Absent | 31737537 | Marschall (2019) Cardiovasc Diagn Ther 9:S292 |
| Ala462Ser | c.1384G>T | DCM | 7.98 x 10^-6^ | 24503780 | Pugh (2014) Genet Med 16:601 |
| Leu471Val | c.1411C>G | LVNC | Absent | 33500567 | Mazzarotto (2021) Genet Med 23:856 |
| Tyr473Cys | c.1418A>G | ACM | Absent | 31956495 | Good (2020) HeartRhythm Case Rep 6:15 |
| **Thr495Met* | *c.1484C>T* | *HCM* | *2.76 x 10^-4^* | *17097056* | *Theis (2006) Biochem Biophys Res Commun 351:896* |
| His518Tyr | c.1552C>T | DCM | 5.57 x 10^-5^ | 31983221 | Mazzarotto (2020) Circulation 141:387 |
| Ile544Leu | c.1630A>C | HCM | 3.98 x 10^-6^ | 31534214 | Lahrouchi (2020) Eur J Hum Genet 28:17 |
| Arg572Trp | c.1714C>T | HCM | 5.66 x 10^-5^ | 27532257 | Walsh (2017) Genet Med 19:192 |
| Glu583Ala | c.1748A>C | HCM | 2.39 x 10^-5^ | 20022194 | Chiu (2010) J Am Coll Cardiol 55:1127 |
| Ile591Val | c.1771A>G | HCM | 3.98 x 10^-6^ | 27532257 | Walsh (2017) Genet Med 19:192 |
| **Asp622Asn* | *c.1864G>A* | *HCM* | *2.90 x 10^-4^* | *31737537* | *Marschall (2019) Cardiovasc Diagn Ther 9:S292* |
| Glu628Gly | c.1883A>G | HCM | Absent | 20022194 | Chiu (2010) J Am Coll Cardiol 55:1127 |
| Glu636Gly | c.1907A>G | DCM | Absent | 32880476 | Verdonschot (2020) Circ Genom Precis Med 13:476 |
| Ile653Thr | c.1958T>C | HCM | Absent | 27532257 | Walsh (2017) Genet Med 19:192 |
| Thr668Arg | c.2003C>G | DCM | 5.17 x 10^-5^ | 27532257 | Walsh (2017) Genet Med 19:192 |
| Ala705Thr | c.2113G>A | DCM | Absent | 28123168 | Higashi (2017) Circ J 81:895 |
| Ala732Thr | c.2194G>A | DCM | 3.18 x 10^-5^ | 32826072 | Peña-Peña (2021) Med Clin (Barc) 156:485 |
| Arg759Thr | c.2276G>C | HCM | Absent | 17097056 | Theis (2006) Biochem Biophys Res Commun 351:896 |
| Asn763Lys | c.2289C>G | DCM | 3.98 x 10^-6^ | 27532257 | Walsh (2017) Genet Med 19:192 |
| His775Tyr | c.2323C>T | DCM | 1.19 x 10^-5^ | 20474083 | Zimmerman (2010) Genet Med 12:268 |
| Arg796Cys | c.2386C>T | HCM | 1.77 x 10^-5^ | 27532257 | Walsh (2017) Genet Med 19:192 |
| Asp826Asn | c.2476G>A | DCM | 7.96 x 10^-6^ | 31983221 | Mazzarotto (2020) Circulation 141:387 |
| Glu849Gly | c.2546A>G | DCM | Absent | 32746448 | Burstein (2021) Pediatr Res 89:1470 PubMed |
| Arg851His | c.2552G>A | DCM | 4.06 x 10^-5^ | 31983221 | Mazzarotto (2020) Circulation 141:387 |
| Arg852Gln | c.2555G>A | HCM | 7.08 x 10^-5^ | 28640247 | Ko (2018) Genet Med 20:69 |
| Asp857His | c.2569G>C | HCM | 2.39 x 10^-5^ | 26573135 | Xu (2015) Sci Rep 5:16609 |
| Gly877Cys | c.2629G>T | DCM | 1.99 x 10^-5^ | 31737537 | Marschall (2019) Cardiovasc Diagn Ther 9:S292 |
| Asp893Gly | c.2678A>G | HCM | 2.12 x 10^-5^ | 32746448 | Burstein (2021) Pediatr Res 89:1470 |

**Supplementary Table 2: *FLNC* missense variants reported in different types of cardiomyopathies**

Abbreviations: DCM, dilated cardiomyopathy; HCM, hypertrophic cardiomyopathy; LVNC, left ventricular noncompaction; RCM, restrictive cardiomyopathy; ACM, arrhythmogenic cardiomyopathy. *variants with a Minor Allele Frequency (MAF) > 1 x 10^−4^. MAF retrieved from the Genome Aggregation Database (gnomAD).

| **Filamin C Missense Variants** | **Nucleotide Change** | **Disease-associated** | **MAF on GnomAD** | **Pubmed ID** | **Reference** |
| --- | --- | --- | --- | --- | --- |
| Phe106Leu | c.318C>G | DCM | 5.04 x 10^-5^ | 27601210 | Reinstein (2016) Eur J Hum Genet 24:1792 |
| Glu110Lys | c.328G>A | DCM | Absent | 32112656 | Verdonschot (2020) Hum Mutat 41:1091 |
| Val123Ala | c.368T>C | HCM | Absent | 25351925 | Valdés-Mas (2014) Nat Commun 5:5326 |
| Val123Met | c.367G>A | DCM | 4.02 x 10^-6^ | 32112656 | Verdonschot (2020) Hum Mutat 41:1091 |
| Asp150Gly | c.449A>G | ACM | 3.59 x 10^-5^ | 32112656 | Verdonschot (2020) Hum Mutat 41:1091 |
| Arg164Trp | c.490C>T | LVNC | 4.01 x 10^-6^ | 31918855 | Liu (2020) Int J Cardiol 302:117 |
| Ala199Pro | c.595G>C | ACM | Absent | 34601126 | Celeghin (2022) Heart Rhythm 19:235 |
| His248Leu | c.743A>T | HCM | Absent | 32112656 | Verdonschot (2020) Hum Mutat 41:1091 |
| Asn290Lys | c.870C>A | HCM | Absent | 25351925 | Valdés-Mas (2014) Nat Commun 5:5326 |
| Asn329Ser | c.986A>G | HCM | 1.20 x 10^-5^ | 28138913 | Restrepo-Cordoba (2017) J Cardiovasc Transl Res 10:35 |
| Ile359Thr | c.1076T>C | HCM | Absent | 30411535 | Cui (2018) Mol Genet Genomic Med 6:1104 |
| Val378Leu | c.1132G>T | HCM | Absent | 30411535 | Cui (2018) Mol Genet Genomic Med 6:1104 |
| Ala427Thr | c.1279G>A | HCM | Absent | 30418145 | Ader (2018) Med Sci (Paris) :39 |
| Arg441Ile | c.1322G>T | DCM | Absent | 32154132 | Xiao (2020) Transl Pediatr 9:21 |
| Pro458Arg | c.1373C>G | ACM | Absent | 34601126 | Celeghin (2022) Heart Rhythm 19:235 |
| Asn475Lys | c.1425C>A | HCM | Absent | 32112656 | Verdonschot (2020) Hum Mutat 41:1091 |
| **Lys492Glu* | *c.1474A>G* | *DCM* | *2.60 x 10^-4^* | *30847666* | *Van Lint (2019) Neth Heart J 27:304* |
| Val628Met | c.1882G>A | HCM | Absent | 30411535 | Cui (2018) Mol Genet Genomic Med 6:1104 |
| Val684Leu | c.2050G>C | HCM | 1.60 x 10^-5^ | 30411535 | Cui (2018) Mol Genet Genomic Med 6:1104 |
| Arg695His | c.2084G>A | HCM | 1.60 x 10^-5^ | 32112656 | Verdonschot (2020) Hum Mutat 41:1091 |
| Pro713Ala | c.2137C>G | ACM | 4.02 x 10^-6^ | 34601126 | Celeghin (2022) Heart Rhythm 19:235 |
| Ile714Thr | c.2141T>C | ACM | 3.19 x 10^-5^ | 31627847 | Hall (2019) Int J Cardiol 307:101 |
| Gly724Ser | c.2170G>A | HCM | 4.06 x 10^-6^ | 30411535 | Cui (2018) Mol Genet Genomic Med 6:1104 |
| Ser792Ile | c.2375G>T | HCM | 6.34 x 10^-6^ | 27574918 | Jaafar (2016) Genet Test Mol Biomarkers 20:674 |
| Val809Met | c.2425G>A | DCM | 1.78 x 10^-5^ | 28436997 | Janin (2017) Clin Genet 92:616 |
| Ile817Thr | c.2450T>C | HCM | 2.50 x 10^-5^ | 29030401 | Cirino (2017) Circ Cardiovasc Genet 10:e001768 |
| Pro863Ser | c.2587C>T | HCM | 4.15 x 10^-6^ | 30411535 | Cui (2018) Mol Genet Genomic Med 6:1104 |
| Glu913Lys | c.2737G>A | HCM | 1.21 x 10^-5^ | 30411535 | Cui (2018) Mol Genet Genomic Med 6:1104 |
| Tyr1042Cys | c.3125A>G | DCM | 3.18 x 10^-5^ | 31931689 | Ramchand (2020) J Am Heart Assoc 9:e013346 |
| Arg1159Trp | c.3475C>T | HCM | 1.78 x 10^-5^ | 30418145 | Ader (2018) Med Sci (Paris) :39 |
| Arg1167Cys | c.3499C>T | HCM | 2.01 x 10^-5^ | 31513939 | Robyns (2020) Eur J Med Genet 63 |
| Ala1186Val | c.3557C>T | RCM | Absent | 29858533 | Kiselev (2018) Hum Mutat 39:9 |
| Ser1194Leu | c.3581C>T | HCM | 4.01 x 10^-6^ | 30418145 | Ader (2018) Med Sci (Paris) :39 |
| Ala1208Val | c.3623C>T | HCM | 9.61 x 10^-5^ | 30411535 | Cui (2018) Mol Genet Genomic Med 6:1104 |
| Thr1227Ile | c.3680C>T | HCM | 3.21 x 10^-5^ | 30418145 | Ader (2018) Med Sci (Paris) :39 |
| Lys1398Glu | c.4192A>G | DCM | Absent | 32659924 | Kolokotronis (2020) J Clin Med 9: |
| Gly1424Val | c.4271G>T | HCM | Absent | 30418145 | Ader (2018) Med Sci (Paris) :39 |
| Tyr1519Cys | c.4556A>G | DCM | 4.30 x 10^-6^ | 32746448 | Burstein (2021) Pediatr Res 89:1470 |
| Ala1539Thr | c.4615G>A | HCM | Absent | 25351925 | Valdés-Mas (2014) Nat Commun 5:5326 |
| **Arg1567Gln* | *c.4700G>A* | *DCM* | *7.42 x 10^-2^* | *28296976* | *Esslinger (2017) PLoS One 12:e0172995* |
| Gly1570Cys | c.4708G>T | HCM | Absent | 31513939 | Robyns (2020) Eur J Med Genet 63: |
| Thr1575Ser | c.4724C>G | ACM | 1.61 x 10^-5^ | 34601126 | Celeghin (2022) Heart Rhythm 19:235 |
| Thr1599Ala | c.4795A>G | HCM | 4.02 x 10^-6^ | 28356264 | Gómez (2017) Circ Cardiovasc Genet 10:e001584 |
| Ser1624Leu | c.4871C>T | RCM | 3.19 x 10^-5^ | 26666891 | Brodehl (2016) Hum Mutat 37:269 |
| Cys1639Tyr | c.4916G>A | RCM | Absent | 32154132 | Xiao (2020) Transl Pediatr 9:21 |
| Thr1664Met | c.4991C>T | LVNC | Absent | 31918855 | Liu (2020) Int J Cardiol 302:117 |
| Ile1666Thr | c.4997T>C | LVNC | Absent | 30418145 | Ader (2018) Med Sci (Paris) :39 |
| Thr1679Lys | c.5036C>A | DCM | 4.01 x 10^-6^ | 32112656 | Verdonschot (2020) Hum Mutat 41:1091 |
| Thr1681Met | c.5042C>T | HCM | 3.92 x 10^-5^ | 28356264 | Gómez (2017) Circ Cardiovasc Genet 10:e001584 |
| Leu1690Phe | c.5068C>T | HCM | Absent | 28356264 | Gómez (2017) Circ Cardiovasc Genet 10:e001584 |
| Pro1709Ser | c.5125C>T | HCM | Absent | 30411535 | Cui (2018) Mol Genet Genomic Med 6:1104 |
| Pro1711Leu | c.5132C>T | HCM | 8.02 x 10^-6^ | 30411535 | Cui (2018) Mol Genet Genomic Med 6:1104 |
| Trp1766Arg | c.5296T>C | HCM | 6.89 x 10^-5^ | 31513939 | Robyns (2020) Eur J Med Genet 63: |
| Ile1882Val | c.5644A>G | ACM | Absent | 31627847 | Hall (2019) Int J Cardiol 307:101 |
| Arg1931Cys | c.5791C>T | DCM | 4.60 x 10^-5^ | 32112656 | Verdonschot (2020) Hum Mutat 41:1091 |
| Ile1937Asn | c.5810T>A | HCM | Absent | 33673806 | Hathaway (2021) BMC Cardiovasc Disord 21:126 |
| Thr1963Met | c.5888C>T | HCM | 3.21 x 10^-5^ | 30411535 | Cui (2018) Mol Genet Genomic Med 6:1104 |
| Ala1979Thr | c.5935G>A | HCM | 1.43 x 10^-5^ | 33455984 | Qin (2021) Int Heart J 62:127 |
| **Ser1985Leu* | *c.5954C>T* | *HCM* | *2.00 x 10^-4^* | *32112656* | *Verdonschot (2020) Hum Mutat 41:1091* |
| Arg1999Gln | c.5996G>A | HCM | 1.61 x 10^-5^ | 27574918 | Jaafar (2016) Genet Test Mol Biomarkers 20:674 |
| Gly2011Arg | c.6031G>A | DCM | Absent | 32659924 | Kolokotronis (2020) J Clin Med 9: |
| Gly2011Glu | c.6032G>A | HCM | Absent | 30418145 | Ader (2018) Med Sci (Paris) :39 |
| Arg2018His | c.6053G>A | HCM | 2.81 x 10^-5^ | 26688388 | Chanavat (2016) Clin Chim Acta 453:80 |
| Gly2034Arg | c.6100G>C | DCM | Absent | 32112656 | Verdonschot (2020) Hum Mutat 41:1091 |
| Gly2039Arg | c.6115G>A | HCM | 7.12 x 10^-6^ | 30418145 | Ader (2018) Med Sci (Paris) :39 |
| Arg2045Gln | c.6134G>A | HCM | 4.28 x 10^-5^ | 26688388 | Chanavat (2016) Clin Chim Acta 453:80 |
| Gln2058Arg | c.6173A>G | ACM | Absent | 31627847 | Hall (2019) Int J Cardiol 307:101 |
| Ala2069Thr | c.6205G>A | HCM | Absent | 32112656 | Verdonschot (2020) Hum Mutat 41:1091 |
| Gly2070Ser | c.6208G>A | DCM, ACM | Absent | 27908349 | Ortiz-Genga (2016) J Am Coll Cardiol 68:2440 |
| Gly2080Arg | c.6238G>C | HCM | Absent | 32037394 | Reuter (2020) Genet Med 22:1015 |
| Ile2109Val | c.6325A>G | ACM | 2.00 x 10^-5^ | 34601126 | Celeghin (2022) Heart Rhythm 19:235 |
| Arg2133Cys | c.6397C>T | HCM | Absent | 30411535 | Cui (2018) Mol Genet Genomic Med 6:1104 |
| Arg2133His | c.6398G>A | HCM | Absent | 25351925 | Valdés-Mas (2014) Nat Commun 5:5326 |
| Arg2140Gln | c.6419G>A | HCM | 2.01 x 10^-5^ | 28356264 | Gómez (2017) Circ Cardiovasc Genet 10:e001584 |
| Gly2151Ser | c.6451G>A | HCM | Absent | 25351925 | Valdés-Mas (2014) Nat Commun 5:5326 |
| Ile2160Phe | c.6478A>T | RCM | Absent | 26666891 | Brodehl (2016) Hum Mutat 37:269 |
| Arg2173His | c.6518G>A | DCM | Absent | 32112656 | Verdonschot (2020) Hum Mutat 41:1091 |
| Arg2180Cys | c.6538C>T | ACM | 2.84 x 10^-5^ | 32112656 | Verdonschot (2020) Hum Mutat 41:1091 |
| Arg2187Cys | c.6559C>T | ACM | 8.09 x 10^-6^ | 32112656 | Verdonschot (2020) Hum Mutat 41:1091 |
| Arg2197Trp | c.6589C>T | HCM | Absent | 28138913 | Restrepo-Cordoba (2017) J Cardiovasc Transl Res 10:35 |
| Lys2260Arg | c.6779A>G | ACM | 4.11 x 10^-5^ | 31627847 | Hall (2019) Int J Cardiol 307:101 |
| Ala2264Thr | c.6790G>A | DCM | 1.07 x 10^-5^ | 32112656 | Verdonschot (2020) Hum Mutat 41:1091 |
| Thr2287Lys | c.6860C>A | HCM | Absent | 32112656 | Verdonschot (2020) Hum Mutat 41:1091 |
| Arg2293Cys | c.6877C>T | DCM | 1.07 x 10^-5^ | 32112656 | Verdonschot (2020) Hum Mutat 41:1091 |
| Gly2294Asp | c.6881G>A | DCM | 4.25 x 10^-6^ | 33726816 | Stranneheim (2021) Genome Med 13:40 |
| Val2297Met | c.6889G>A | RCM | 4.22 x 10^-6^ | 29212899 | Tucker (2017) Circ Cardiovasc Genet 10:e001780 |
| Pro2298Leu | c.6893C>T | RCM | Absent | 30260051 | Schubert (2018) Hum Mutat 39:2083 |
| Pro2298Ser | c.6892C>T | HCM | Absent | 28356264 | Gómez (2017) Circ Cardiovasc Genet 10:e001584 |
| Gly2299Ser | c.6895G>A | HCM | Absent | 30418145 | Ader (2018) Med Sci (Paris) :39 |
| Pro2301Ala | c.6901C>G | HCM | Absent | 28356264 | Gómez (2017) Circ Cardiovasc Genet 10:e001584 |
| Pro2301Leu | c.6902C>T | RCM | Absent | 30919686 | Roldán-Sevilla (2019) Circ Genom Precis Med 12:e002388 |
| Pro2308Leu | c.6923C>T | HCM | 4.14 x 10^-5^ | 30418145 | Ader (2018) Med Sci (Paris) :39 |
| His2315Asn | c.6943C>A | HCM | Absent | 25351925 | Valdés-Mas (2014) Nat Commun 5:5326 |
| Lys2316Arg | c.6947A>G | HCM | Absent | 31513939 | Robyns (2020) Eur J Med Genet 63: |
| Arg2318Trp | c.6952C>T | HCM | 8.19 x 10^-6^ | 28356264 | Gómez (2017) Circ Cardiovasc Genet 10:e001584 |
| Ala2344Thr | c.7030G>A | HCM | 8.02 x 10^-6^ | 30411535 | Cui (2018) Mol Genet Genomic Med 6:1104 |
| Ile2359Thr | c.7076T>C | HCM | Absent | 30418145 | Ader (2018) Med Sci (Paris) :39 |
| Val2375Leu | c.7123G>C | HCM | Absent | 30418145 | Ader (2018) Med Sci (Paris) :39 |
| Val2375Phe | c.7123G>T | HCM | Absent | 28356264 | Gómez (2017) Circ Cardiovasc Genet 10:e001584 |
| Arg2410Cys | c.7228C>T | HCM | 2.41 x 10^-5^ | 30418145 | Ader (2018) Med Sci (Paris) :39 |
| Gln2417Pro | c.7250A>C | HCM | Absent | 30418145 | Ader (2018) Med Sci (Paris) :39 |
| Leu2421His | c.7262T>A | HCM | Absent | 31006259 | Norrish (2019) Circulation 140:184 |
| **Ala2430Val* | *c.7289C>T* | *HCM* | *1.01 x 10^-4^* | *25351925* | *Valdés-Mas (2014) Nat Commun 5:5326* |
| His2476Arg | c.7427A>G | HCM | Absent | 31513939 | Robyns (2020) Eur J Med Genet 63: |
| Gly2484Ser | c.7450G>A | DCM | 2.80 x 10^-5^ | 32112656 | Verdonschot (2020) Hum Mutat 41:1091 |
| Arg2495His | c.7484G>A | HCM | 4.01 x 10^-6^ | 30418145 | Ader (2018) Med Sci (Paris) :39 |
| Pro2505Leu | c.7514C>T | HCM | Absent | 30411535 | Cui (2018) Mol Genet Genomic Med 6:1104 |
| Asp2551Gly | c.7652A>G | DCM | 8.03 x 10^-6^ | 32112656 | Verdonschot (2020) Hum Mutat 41:1091 |
| Tyr2563Cys | c.7688A>G | RCM | Absent | 30260051 | Schubert (2018) Hum Mutat 39:2083 |
| Gly2594Ala | c.7781G>C | HCM | Absent | 26688388 | Chanavat (2016) Clin Chim Acta 453:80 |
| Ser2618Phe | c.7853C>T | HCM | Absent | 32112656 | Verdonschot (2020) Hum Mutat 41:1091 |

**Supplementary Table 3: *TTN* missense variants in the Z-disk region (first 900 amino acids) reported in different types of cardiomyopathies**

Abbreviations: DCM, dilated cardiomyopathy; HCM, hypertrophic cardiomyopathy; LVNC, left ventricular noncompaction. *variants with a Minor Allele Frequency (MAF) > 1 x 10^−4^. MAF retrieved from the Genome Aggregation Database (gnomAD).

| **Titin Missense Variants** | **Nucleotide Change** | **Disease-associated** | **MAF on GnomAD** | **Pubmed ID** | **Reference** |
| --- | --- | --- | --- | --- | --- |
| Pro6Leu | c.17C>T | DCM | 1.20 x 10^-5^ | 31983221 | Mazzarotto (2020) Circulation 141:387 |
| Val17Leu | c.49G>T | HCM | Absent | Absent | Micheu (2022) Romanian Journal of Cardiology 31:3 |
| Gly43Ala | c.128G>C | DCM | Absent | 31983221 | Mazzarotto (2020) Circulation 141:387 |
| Val45Leu | c.133G>C | DCM | 2.83 x 10^-5^ | 31983221 | Mazzarotto (2020) Circulation 141:387 |
| Val54Met | c.160G>A | DCM | 4.25 x 10^-5^ | 11846417 | Itoh-Satoh (2002) Biochem Biophys Res Commun 291:385 |
| Asp60Asn | c.178G>A | DCM | 1.59 x 10^-5^ | 31983221 | Mazzarotto (2020) Circulation 141:387 |
| Ala178Asp | c.533C>A | DCM, LVNC | Absent | 27625337 | Hastings (2016) Circ Cardiovasc Genet 9:426 |
| Arg220Gln | c.659G>A | LVNC | 1.42 x 10^-5^ | 30847666 | van Lint (2019) Neth Heart J 27:304 |
| Thr253Ile | c.758C>T | HCM | Absent | 28771489 | Mademont-Soler (2017) PLoS One 12:e0181465 |
| Ser306Pro | c.916T>C | DCM | Absent | 31983221 | Mazzarotto (2020) Circulation 141:387 |
| Thr334Ala | c.1000A>G | DCM | 3.98 x 10^-6^ | 31983221 | Mazzarotto (2020) Circulation 141:387 |
| Ile443Met | c.1329C>G | DCM | 3.18 x 10^-5^ | 31983221 | Mazzarotto (2020) Circulation 141:387 |
| Ala450Val | c.1349C>T | DCM | 3.98 x 10^-6^ | 32746448 | Burstein (2021) Pediatr Res 89:1470 |
| Gln547Arg | c.1640A>G | DCM | 6.75 x 10^-5^ | 31983221 | Mazzarotto (2020) Circulation 141:387 |
| Gly721Arg | c.2161G>A | DCM | Absent | 31983221 | Mazzarotto (2020) Circulation 141:387 |
| Arg740Leu | c.2219G>T | HCM | Absent | 10462489 | Satoh (1999) Biochem Biophys Res Commun 262:411 |
| Ala743Val | c.2228C>T | DCM | Absent | 11846417 | Itoh-Satoh (2002) Biochem Biophys Res Commun 291:385 |
| **Gln770His* | *c.2310G>T* | *DCM* | *1.10 x 10^-4^* | *31983221* | *Mazzarotto (2020) Circulation 141:387* |
| His786Gln | c.2358C>G | DCM | 7.44 x 10^-5^ | 31983221 | Mazzarotto (2020) Circulation 141:387 |
| Ala840Ser | c.2518G>T | HCM | Absent | Absent | Micheu (2022) Romanian Journal of Cardiology 31:3 |
| Thr863Ala | c.2587A>G | DCM | 3.98 x 10^-6^ | 31983221 | Mazzarotto (2020) Circulation 141:387 |

**Supplementary Table 4: *CSPR3* missense variants reported in different types of cardiomyopathies**

Abbreviations: DCM, dilated cardiomyopathy; HCM, hypertrophic cardiomyopathy. *variants with a Minor Allele Frequency (MAF) > 1 x 10^−4^. MAF retrieved from the Genome Aggregation Database (gnomAD).

| ***CSPR3***  **Missense Variants** | **Nucleotide Change** | **Disease-associated** | **MAF on GnomAD** | **Pubmed ID** | **Reference** |
| --- | --- | --- | --- | --- | --- |
| Met1Thr | c.2T>C | DCM | Absent | 29886034 | Shakeel (2018) Gene 673:134 |
| Asn3Lys | c.9C>G | DCM | Absent | 32880476 | Verdonschot (2020) Circ Genom Precis Med 13:476 |
| **Trp4Arg* | *c.10T>C* | *DCM* | *2.28 x 10^-3^* | *12507422* | *Knoll (2002) Cell 111:943* |
| **Gly6Arg* | *c.16G>A* | *DCM* | *1.06 x 10^-4^* | *31983221* | *Mazzarotto (2020) Circulation 141:387* |
| Thr16Ser | c.46A>T | DCM | Absent | 31919335 | Lipari (2020) Pol Arch Intern Med 130:89 |
| His31Gln | c.93C>G | HCM | Absent | 28790153 | Burns (2017) Circ Cardiovasc Genet 10:e001666 |
| Lys32Lys | c.96G>A | DCM | Absent | 31919335 | Lipari (2020) Pol Arch Intern Med 130:89 |
| His36Tyr | c.106C>T | DCM | Absent | 31983221 | Mazzarotto (2020) Circulation 141:387 |
| Leu44Pro | c.131T>C | HCM | 1.42 x 10^-5^ | 12642359 | Geier (2003) Circulation 107:1390 |
| Ser46Arg | c.136A>C | HCM | 2.83 x 10^-5^ | 18505755 | Geier (2008) Hum Mol Genet 17:2753 |
| Thr47Met | c.140C>T | HCM | 2.39 x 10^-5^ | 27532257 | Walsh (2017) Genet Med 19:192 |
| Ala50Thr | c.148G>A | DCM | 3.90 x 10^-5^ | 20474083 | Zimmerman (2010) Genet Med 12:268 |
| Ala51Asp | c.152C>A | HCM | 2.39 x 10^-5^ | 27532257 | Walsh (2017) Genet Med 19:192 |
| Ser54Leu | c.161C>T | HCM | 1.19 x 10^-5^ | 30847666 | van Lint (2019) Neth Heart J 27:304 |
| Tyr57Ser | c.170A>C | HCM | Absent | 22429680 | Santos (2012) BMC Med Genet 13:17 |
| Cys58Gly | c.172T>G | HCM | Absent | 12642359 | Geier (2003) Circulation 107:1390 |
| Arg64Cys | c.190C>T | HCM | 3.89 x 10^-5^ | 16352453 | Bos (2006) Mol Genet Metab 88:78 |
| Arg64His | c.191G>A | HCM | 2.12 x 10^-5^ | 27532257 | Walsh (2017) Genet Med 19:192 |
| Arg64Leu | c.191G>T | HCM | Absent | 31513939 | Robyns (2020) Eur J Med Genet 63: |
| Tyr66Cys | c.197A>G | HCM | 7.96 x 10^-6^ | 16352453 | Bos (2006) Mol Genet Metab 88:78 |
| Lys69Arg | c.206A>G | DCM | 1.99 x 10^-5^ | 14567970 | Mohapatra (2003) Mol Genet Metab 80:207 |
| Gly70Trp | c.208G>T | HCM | 3.58 x 10^-5^ | 33297573 | Micheu (2020) Diagnostics (Basel) 10: |
| Arg70Trp | C.208C>T | HCM | Absent | 16352453 | Bos (2006) Mol Genet Metab 88:78 |
| Gly72Arg | c.214G>A | DCM | 2.48 x 10^-5^ | 19412328 | Hershberger (2008) Clin Transl Sci 1:21 |
| Gly78Val | c.233G>T | DCM | Absent | 31919335 | Lipari (2020) Pol Arch Intern Med 130:89 |
| Thr84Met | c.251C>T | HCM | 1.19 x 10^-5^ | 31006259 | Norrish (2019) Circulation 140:184 |
| Gly89Cys | c.265G>T | DCM | 1.77 x 10^-5^ | 32880476 | Verdonschot (2020) Circ Genom Precis Med 13:476 |
| Gln91Glu | c.271C>G | HCM | 3.99 x 10^-6^ | 32815737 | Pua (2020) Circ Genom Precis Med 13:424 |
| Gln91Leu | c.272A>T | HCM | 1.42 x 10^-5^ | 16352453 | Bos (2006) Mol Genet Metab 88:78 |
| **Arg100His* | *c.299G>A* | *HCM* | *1.32 x 10^-3^* | *19035361* | *Andersen (2009) Hum Mutat 30:363* |
| Gly115Glu | c.344G>A | HCM | 7.96 x 10^-6^ | 30847666 | Lint (2019) Neth Heart J 27:304 |
| Glu118Glu | c.354G>A | DCM | Absent | 31919335 | Lipari (2020) Pol Arch Intern Med 130:89 |
| Val127Ile | c.379G>A | HCM | 3.58 x 10^-5^ | 27532257 | Walsh (2017) Genet Med 19:192 |
| Trp140Cys | c.420G>C | DCM | Absent | 31919335 | Lipari (2020) Pol Arch Intern Med 130:89 |
| Arg146Cys | c.436C>T | HCM | 1.19 x 10^-5^ | 28790153 | Burns (2017) Circ Cardiovasc Genet 10:e001666 |
| Arg146His | c.437G>A | HCM | 1.99 x 10^-5^ | 28790153 | Burns (2017) Circ Cardiovasc Genet 10:e001666 |
| Cys150Tyr | c.449G>A | HCM | 3.98 x 10^-6^ | 33035702 | Salazar-Mendiguchía (2020) Eur J Med Genet 63: |
| Asn158Asp | c.472A>G | HCM | 7.95 x 10^-6^ | 30847666 | Van Lint (2019) Neth Heart J 27:304 |
| Cys171Gly | c.511T>G | HCM | 3.98 x 10^-6^ | 28790153 | Burns (2017) Circ Cardiovasc Genet 10:e001666 |
| Tyr172His | c.514T>C | HCM | Absent | 30847666 | van Lint (2019) Neth Heart J 27:304 |
| Thr179Ala | c.535A>G | DCM | 1.41 x 10^-5^ | 27532257 | Walsh (2017) Genet Med 19:192 |
| Val190Leu | c.568G>T | HCM | 4.60 x 10^-5^ | 31737537 | Marschall (2019) Cardiovasc Diagn Ther 9:S292 |

**Supplementary Table 5: *TCAP* missense variants reported in different types of cardiomyopathies**

Abbreviations: DCM, dilated cardiomyopathy; HCM, hypertrophic cardiomyopathy. *variants with a Minor Allele Frequency (MAF) > 1 x 10^−4^. MAF retrieved from the Genome Aggregation Database (gnomAD).

| **Telethonin Missense Variants** | **Nucleotide Change** | **Disease-associated** | **MAF on GnomAD** | **Pubmed ID** | **Reference** |
| --- | --- | --- | --- | --- | --- |
| Arg18Gln | c.53G>A | DCM | 2.39 x 10^-5^ | 19412328 | Hershberger (2008) Clin Transl Sci 1:21 |
| Arg18Trp | c.52C>T | DCM | 1.59 x 10^-5^ | 31983221 | Mazzarotto (2020) Circulation 141:387 |
| Ala23Glu | c.68C>A | HCM | Absent | 33297573 | Micheu (2020) Diagnostics (Basel) 10:1061 |
| Glu49Lys | c.145G>A | DCM | 3.70 x 10^-5^ | 19412328 | Hershberger (2008) Clin Transl Sci 1:21 |
| Cys57Trp | c.171C>G | HCM | 1.66 x 10^-5^ | 32565061 | Toste (2020) Rev Port Cardiol 39:317 |
| Arg70Trp | c.208C>T | HCM | 4.62 x 10^-5^ | 16352453 | Bos (2006) Mol Genet Metab 88:78 |
| Arg87Gln | c.260G>A | DCM | 4.12 x 10^-6^ | 12507422 | Knoll (2002) Cell 111:943 |
| Pro90Leu | c.269C>T | HCM | 4.10 x 10^-6^ | 16352453 | Bos (2006) Mol Genet Metab 88:78 |
| **Glu105Gln* | *c.313G>C* | *DCM* | *5.10 x 10^-4^* | *30871747* | *Sousa (2019) Rev Port Cardiol 38:129* |
| **Arg106Cys* | *c.316C>T* | *HCM* | *2.19 x 10^-2^* | *19035361* | *Andersen (2009) Hum Mutat 30:363* |
| Arg130Cys | c.388C>T | DCM | 5.69 x 10^-5^ | 27532257 | Walsh (2017) Genet Med 19:192 |
| Glu132Gln | c.394G>C | DCM | 4.01 x 10^-6^ | 15582318 | Hayashi (2004) J Am Coll Cardiol 44:2192 |
| Thr137Ile | c.410C>T | HCM | Absent | 15582318 | Hayashi (2004) J Am Coll Cardiol 44:2192 |
| Pro142Arg | c.425C>G | HCM | 4.04 x 10^-6^ | 33658040 | Chung (2021) J Cardiovasc Magn Reson 23:18 |
| Gly150Ser | c.448G>A | DCM | 5.79 x 10^-5^ | 31983221 | Mazzarotto (2020) Circulation 141:387 |
| **Arg153His* | *c.458G>A* | *HCM* | *2.37 x 10^-4^* | *15582318* | *Hayashi (2004) J Am Coll Cardiol 44:2192* |
| Arg158Cys | c.472C>T | DCM | 8.28 x 10^-6^ | 24037902 | Hirtle-Lewis (2013) Clin Cardiol 36:628 |
| Arg158Ser | c.472C>A | DCM | Absent | 26084686 | Akinrinade (2015) Eur Heart J 36:2327 |
| Gln165Glu | c.493C>G | DCM | Absent | 27532257 | Walsh (2017) Genet Med 19:192 |

**Supplementary Table 6: *MYPN* missense variants reported in different types of cardiomyopathies**

Abbreviations: DCM, dilated cardiomyopathy; HCM, hypertrophic cardiomyopathy; LVNC, left ventricular noncompaction. *variants with a Minor Allele Frequency (MAF) > 1 x 10^−4^. MAF retrieved from the Genome Aggregation Database (gnomAD).

| **Myopalladin Missense Variants** | **Nucleotide Change** | **Disease-associated** | **MAF on GnomAD** | **Pubmed ID** | **Reference** |
| --- | --- | --- | --- | --- | --- |
| Met1Ile | c.3G>A | DCM | Absent | 29095814 | Hu (2018) Genet Med 20:1045 |
| **Tyr20Cys* | *c.59A>G* | *DCM, HCM* | *9.34 x 10^-4^* | *22286171* | *Purevjav (2012) Hum Mol Genet 21:2039* |
| **Ala22Gly* | *c.65C>G* | *HCM* | *2.55 x 10^-4^* | *32746448* | *Burstein (2021) Pediatr Res 89:1470* |
| Pro47Leu | c.140C>T | DCM | 6.72 x 10^-5^ | 31983221 | Mazzarotto (2020) Circulation 141:387 |
| Arg137Ser | c.411G>C | HCM | 3.89 x 10^-5^ | 30165862 | Lu (2018) J Transl Med 16:241 |
| Lys153Arg | c.458A>G | HCM | 9.55 x 10^-5^ | 22286171 | Purevjav (2012) Hum Mol Genet 21:2039 |
| Asp156Glu | c.468C>G | DCM | Absent | 32041989 | Zhang (2020) Sci Rep 10:2226 |
| Ile213Val | c.637A>G | DCM | Absent | 22286171 | Purevjav (2012) Hum Mol Genet 21:2039 |
| Ala217Glu | c.650C>A | HCM | 3.98 x 10^-6^ | 22286171 | Purevjav (2012) Hum Mol Genet 21:2039 |
| Ala265Pro | c.793G>C | HCM | Absent | 22286171 | Purevjav (2012) Hum Mol Genet 21:2039 |
| Pro269Leu | c.806C>T | DCM | 4.00 x 10^-6^ | 26383716 | Hazebroek (2015) J Am Coll Cardiol 66:1313 |
| Arg278Gly | c.832A>G | HCM | 3.99 x 10^-6^ | 30165862 | Lu (2018) J Transl Med 16:241 |
| Arg338Cys | c.1012C>T | HCM | 6.37 x 10^-5^ | 33297573 | Micheu (2020) Diagnostics (Basel) 10: |
| Tyr339Phe | c.1016A>T | DCM | Absent | 22286171 | Purevjav (2012) Hum Mol Genet 21:2039 |
| Val361Phe | c.1081G>T | HCM | 1.59 x 10^-5^ | 30847666 | van Lint (2019) Neth Heart J 27:304 |
| Ser366Pro | c.1096T>C | DCM | 2.39 x 10^-5^ | 31983221 | Mazzarotto (2020) Circulation 141:387 |
| Arg409Cys | c.1225C>T | DCM | 7.96 x 10^-6^ | 25448463 | Chami (2014) Can J Cardiol 30:1655 |
| **Val410Ala* | *c.1229T>C* | *HCM* | *1.67 x 10^-4^* | *22286171* | *Purevjav (2012) Hum Mol Genet 21:2039* |
| **Arg489Gln* | *c.1466G>A* | *DCM* | *1.08 x 10^-4^* | *30847666* | *van Lint (2019) Neth Heart J 27:304* |
| Ala492Val | c.1475C>T | DCM | 5.59 x 10^-5^ | 31983221 | Mazzarotto (2020) Circulation 141:387 |
| Val532Met | c.1594G>A | LVNC | 2.81 x 10^-5^ | 28798025 | Miszalski-Jamka (2017) Circ Cardiovasc Genet 10:e001763 |
| Gly542Arg | c.1624G>A | LVNC | 1.06 x 10^-5^ | 28798025 | Miszalski-Jamka (2017) Circ Cardiovasc Genet 10:e001763 |
| Ala611Thr | c.1831G>A | DCM | Absent | 22286171 | Purevjav (2012) Hum Mol Genet 21:2039 |
| Gln629Pro | c.1886A>C | DCM | Absent | 31983221 | Mazzarotto (2020) Circulation 141:387 |
| Glu630Lys | c.1888G>A | DCM | 7.07 x 10^-6^ | 26458567 | Zhao (2015) Int J Mol Med 36:1479 |
| Leu677Trp | c.2030T>G | LVNC | Absent | 28798025 | Miszalski-Jamka (2017) Circ Cardiovasc Genet 10:e001763 |
| Pro680Leu | c.2039C>T | DCM | Absent | 31983221 | Mazzarotto (2020) Circulation 141:387 |
| Ser684Cys | c.2051C>G | DCM | 2.39 x 10^-5^ | 31983221 | Mazzarotto (2020) Circulation 141:387 |
| Pro713Thr | c.2137C>A | DCM | Absent | 30847666 | van Lint (2019) Neth Heart J 27:304 |
| Leu720Trp | c.2159T>G | DCM | 7.95 x 10^-6^ | 31983221 | Mazzarotto (2020) Circulation 141:387 |
| Thr730Arg | c.2189C>G | HCM | Absent | 31513939 | Robyns (2020) Eur J Med Genet 63: |
| **Pro743Leu* | *c.2228C>T* | *DCM* | *2.51 x 10^-4^* | *32746448* | *Burstein (2021) Pediatr Res 89:1470* |
| Gln752Glu | c.2254C>G | DCM | Absent | 31737537 | Marschall (2019) Cardiovasc Diagn Ther 9:S292 |
| Val765Ala | c.2294T>C | DCM | 7.96 x 10^-6^ | 31737537 | Marschall (2019) Cardiovasc Diagn Ther 9:S292 |
| Arg810Cys | c.2428C>T | HCM | 1.99 x 10^-5^ | 30847666 | van Lint (2019) Neth Heart J 27:304 |
| Pro826Thr | c.2476C>A | HCM | Absent | 30206291 | Inagaki (2018) J Hum Genet 63:1273 |
| Pro835Thr | c.2503C>A | DCM | Absent | 31983221 | Mazzarotto (2020) Circulation 141:387 |
| Pro841Thr | c.2521C>A | HCM | Absent | 22286171 | Purevjav (2012) Hum Mol Genet 21:2039 |
| Ser854Phe | c.2561C>T | HCM | 1.78 x 10^-5^ | 31737537 | Marschall (2019) Cardiovasc Diagn Ther 9:S292 |
| Asn881Ile | c.2642A>T | DCM | 3.54 x 10^-5^ | 32880476 | Verdonschot (2020) Circ Genom Precis Med 13:476 |
| Ala882Thr | c.2644G>A | DCM | 1.77 x 10^-5^ | 22286171 | Purevjav (2012) Hum Mol Genet 21:2039 |
| Glu909Ala | c.2726A>C | DCM | Absent | 32880476 | Verdonschot (2020) Circ Genom Precis Med 13:476 |
| Phe954Leu | c.2862C>A | DCM | Absent | 22286171 | Purevjav (2012) Hum Mol Genet 21:2039 |
| Arg955Gln | c.2864G>A | DCM | 4.60 x 10^-5^ | 31983221 | Mazzarotto (2020) Circulation 141:387 |
| **Arg955Trp* | *c.2863C>T* | *DCM* | *4.71 x 10^-4^* | *22892539* | *Meyer (2013) Eur J Hum Genet 21:294* |
| Pro961Leu | c.2882C>T | DCM | Absent | 22892539 | Meyer (2013) Eur J Hum Genet 21:294 |
| Cys1002Trp | c.3006C>G | HCM | Absent | 31513939 | Robyns (2020) Eur J Med Genet 63: |
| **Arg1042Cys* | *c.3124C>T* | *LVNC* | *4.00 x 10^-4^* | *28798025* | *Miszalski-Jamka (2017) Circ Cardiovasc Genet 10:e001763* |
| Arg1069Cys | c.3205C>T | HCM | 1.19 x 10^-5^ | 33658040 | Chung (2021) J Cardiovasc Magn Reson 23:18 |
| Arg1088His | c.3263G>A | DCM | 3.98 x 10^-6^ | 18006477 | Duboscq-Bidot (2008) Cardiovasc Res 77:118 |
| Pro1100Leu | c.3299C>T | HCM | 3.19 x 10^-5^ | 33658040 | Chung (2021) J Cardiovasc Magn Reson 23:18 |
| **Pro1112Leu* | *c.3335C>T* | *DCM* | *3.06 x 10^-3^* | *18006477* | *Duboscq-Bidot (2008) Cardiovasc Res 77:118* |
| Ala1148Thr | c.3442G>A | DCM | 1.41 x 10^-5^ | 25163546 | Haas (2015) Eur Heart J 36:1123 |
| Gly1153Arg | c.3457G>A | LVNC | 1.19 x 10^-5^ | 30471092 | Richard (2019) Clin Genet 95:356 |
| **Leu1161Ile* | *c.3481C>A* | *HCM* | *3.58 x 10^-3^* | *22286171* | *Purevjav (2012) Hum Mol Genet 21:2039* |
| Val1195Met | c.3583G>A | DCM | Absent | 18006477 | Duboscq-Bidot (2008) Cardiovasc Res 77:118 |
| Arg1218Ser | c.3654G>C | DCM | 7.97 x 10^-6^ | 28416588 | Dal Ferro (2017) Heart 103:1704 |
| Gly1253Arg | c.3757G>C | DCM | Absent | 31983221 | Mazzarotto (2020) Circulation 141:387 |
| Val1255Met | c.3763G>A | DCM | 5.57 x 10^-5^ | 32826072 | Peña-Peña (2021) Med Clin (Barc) 156:485 |
| Ala1259Thr | c.3775G>A | DCM | Absent | 31983221 | Mazzarotto (2020) Circulation 141:387 |
| Ala1265Pro | c.3793G>C | HCM | Absent | 22286171 | Purevjav (2012) Hum Mol Genet 21:2039 |
| Arg1283His | c.3848G>A | HCM | 3.89 x 10^-5^ | 28427417 | Chen (2017) J Transl Med 15:78 |
| Thr1304Met | c.3911C>T | DCM | 1.77 x 10^-5^ | 32880476 | Verdonschot (2020) Circ Genom Precis Med 13:476 |
| Met1305Val | c.3913A>G | DCM | 6.01 x 10^-5^ | 28416588 | Dal Ferro ( 2017) Heart 103:1704 |
| **Arg1312Trp* | *c.3934C>T* | *DCM* | *2.48 x 10^-4^* | *30847666* | *van Lint (2019) Neth Heart J 27:304* |
| Leu1320Pro | c.3959T>C | LVNC | 7.96 x 10^-6^ | 28798025 | Miszalski-Jamka (2017) Circ Cardiovasc Genet 10:e001763 |

**Supplementary Table 7: *NEBL* missense variants reported in different types of cardiomyopathies**

Abbreviations: DCM, dilated cardiomyopathy; HCM, hypertrophic cardiomyopathy; LVNC, left ventricular noncompaction. *variants with a Minor Allele Frequency (MAF) > 1 x 10^−4^. MAF retrieved from the Genome Aggregation Database (gnomAD).

| **Nebulette Missense Variants** | **Nucleotide Change** | **Disease-associated** | **MAF on GnomAD** | **Pubmed ID** | **Reference** |
| --- | --- | --- | --- | --- | --- |
| Val5Ala | c.14T>C | DCM | 6.56 x 10^-5^ | 32746448 | Burstein (2021) Pediatr Res 89:1470 |
| **Lys60Asn* | *c.180G>C* | *DCM* | *4.00 x 10^-3^* | *20951326* | *Purevjav (2010) J Am Coll Cardiol 56:1493* |
| Arg104Trp | c.310C>T | DCM | 9.15 x 10^-5^ | 32746448 | Burstein (2021) Pediatr Res 89:1470 |
| Gln128Arg | c.383A>G | DCM | 2.83 x 10^-5^ | 20951326 | Purevjav (2010) J Am Coll Cardiol 56:1493 |
| His130Pro | c.389A>C | HCM | Absent | 30165862 | Lu (2018) J Transl Med 16:241 |
| His171Arg | c.512A>G | HCM | 3.98 x 10^-6^ | 27186169 | Perrot (2016) Arch Med Sci 12:263 |
| Thr172Lys | c.515C>A | DCM | 1.59 x 10^-5^ | 30165862 | Lu (2018) J Transl Med 16:241 |
| **Ala175Thr* | *c.523G>A* | *DCM* | *1.10 x 10^-4^* | *27186169* | *Perrot (2016) Arch Med Sci 12:263* |
| **Gln187His* | *c.561G>C* | *HCM* | *1.63 x 10^-4^* | *32344918* | *Gao (2020) Int J Mol Sci 21:3040* |
| **Gly202Arg* | *c.604G>A* | *DCM* | *2.03 x 10^-3^* | *20951326* | *Purevjav (2010) J Am Coll Cardiol 56:1493* |
| Ser296Gly | c.886A>G | LVNC | 3.98 x 10^-6^ | 33500567 | Mazzarotto (2021) Genet Med 23:856 |
| Asn330Ser | c.989A>G | LVNC | Absent | 33500567 | Mazzarotto (2021) Genet Med 23:856 |
| Met351Val | c.1051A>G | DCM | 3.98 x 10^-6^ | 34302607 | Xiao (2021) Sci China Life Sci : |
| Phe354Leu | c.1060T>C | HCM | 3.98 x 10^-6^ | 30206291 | Inagaki (2018) J Hum Genet 63:1273 |
| **Asp378His* | *c.1132G>C* | *DCM* | *6.51 x 10^-2^* | *34302607* | *Xiao (2021) Sci China Life Sci :* |
| Ala477Pro | c.1429G>C | DCM | 2.84 x 10^-5^ | 30165862 | Lu (2018) J Transl Med 16:241 |
| **Ile480Thr* | *c.1439T>C* | *DCM* | *1.44 x 10^-4^* | *31737537* | *Marschall (2019) Cardiovasc Diagn Ther 9:S292* |
| Gln581Arg | c.1742A>G | DCM | Absent | 27186169 | Perrot (2016) Arch Med Sci 12:263 |
| **Ala592Glu* | *c.1775C>A* | *DCM* | *3.73 x 10^-4^* | *20951326* | *Purevjav (2010) J Am Coll Cardiol 56:1493* |
| **Arg613Gln* | *c.1838G>A* | *DCM* | *2.41 x 10^-4^* | *32746448* | *Burstein (2021) Pediatr Res 89:1470* |
| Ala726Thr | c.2176G>A | LVNC | Absent | 33500567 | Mazzarotto (2021) Genet Med 23:856 |
| Ser747Leu | c.2240C>T | DCM | 8.50 x 10^-5^ | 27186169 | Perrot (2016) Arch Med Sci 12:263 |
| Gln814Arg | c.2441A>G | DCM | 3.98 x 10^-6^ | 28416588 | Dal Ferro (2017) Heart 103:1704 |
| Arg845Cys | c.2533C>T | ACM | 4.61 x 10^-5^ | 30385303 | Chen (2019) Gene 687:82 |
| Ser863Cys | c.2588C>G | LVNC | 1.99 x 10^-5^ | 33500567 | Mazzarotto (2021) Genet Med 23:856 |
| Pro916Leu | c.2747C>T | LVNC | 6.77 x 10^-5^ | 27186169 | Perrot (2016) Arch Med Sci |

**Supplementary Table 8: *NEXN* missense variants reported in different types of cardiomyopathies**

Abbreviations: DCM, dilated cardiomyopathy; HCM, hypertrophic cardiomyopathy; LVNC, left ventricular noncompaction. *variants with a Minor Allele Frequency (MAF) > 1 x 10^−4^. MAF retrieved from the Genome Aggregation Database (gnomAD).

| **Nexilin Missense Variants** | **Nucleotide Change** | **Disease-associated** | **MAF on GnomAD** | **Pubmed ID** | **Reference** |
| --- | --- | --- | --- | --- | --- |
| Ser15Tyr | c.44C>A | HCM | Absent | 33297573 | Micheu (2020) Diagnostics (Basel) 10:1061 |
| Val18Ala | c.53T>C | DCM | 2.41 x 10^-5^ | 28416588 | Dal Ferro (2017) Heart 103:1704 |
| Glu53Lys | c.157G>A | LVNC | 3.93 x 10^-5^ | 28798025 | Miszalski-Jamka (2017) Circ Cardiovasc Genet 10:e001763 |
| Ile74Leu | c.220A>C | LVNC | Absent | 33500567 | Mazzarotto (2021) Genet Med 23:856 |
| Glu83Asp | c.249G>C | LVNC | 4.01 x 10^-6^ | 33500567 | Mazzarotto (2021) Genet Med 23:856 |
| Glu110Gln | c.328G>C | DCM | 1.07 x 10-5 | 25163546 | Hass (2015) Eur Heart J 36:18 |
| Gln131Arg | c.392A>G | HCM | 2.85 x 10^-5^ | 27532257 | Walsh (2017) Genet Med 19:192 |
| Gln131Glu | c.391C>G | HCM | 4.01 x 10^-6^ | 20970104 | Wang (2010) Am J Hum Genet 87:687 |
| **Arg196Cys* | *c.586C>T* | *DCM* | *1.07 x 10^-4^* | *28333919* | *Bagnall (2017) Genet Med 19:1127* |
| Gly157Val | c.470G>T | DCM | Absent | 25163546 | Hass (2015) Eur Heart J 36:18 |
| Arg256Gln | c.767G>A | HCM | 1.20 x 10^-5^ | 33658040 | Chung (2021) J Cardiovasc Magn Reson 23:18 |
| Lys278Arg | c.833A>G | DCM | Absent | 31983221 | Mazzarotto (2020) Circulation 141:387 |
| **Arg279Cys* | *c.835C>T* | *HCM* | *5.04 x 10^-4^* | *20970104* | *Wang (2010) Am J Hum Genet 87:687* |
| Arg279Ser | c.835C>A | DCM | 1.61 x 10^-5^ | 32041989 | Zhang (2020) Sci Rep 10:2226 |
| **Arg286Trp* | *c.856C>T* | *LVNC* | *1.25 x 10^-4^* | *28798025* | *Miszalski-Jamka (2017) Circ Cardiovasc Genet 10:e001763* |
| Glu291Lys | c.871G>A | HCM | 2.43 x 10^-5^ | 28790153 | Burns (2017) Circ Cardiovasc Genet 10:e001666 |
| Asp292Asn | c.874G>A | DCM | 4.06 x 10^-6^ | 30847666 | van Lint (2019) Neth Heart J 27:304 |
| Tyr305Cys | c.914A>G | DCM | 8.10 x 10^-6^ | 31983221 | Mazzarotto (2020) Circulation 141:387 |
| **Glu332Ala* | *c.995A>C* | *HCM* | *4.07 x 10^-3^* | *26265630* | *Wilson (2015) Circ Res 117:603* |
| Ala333Thr | c.997G>A | DCM | Absent | 31983221 | Mazzarotto (2020) Circulation 141:387 |
| Asp355His | c.1063G>C | HCM | Absent | 28790153 | Burns (2017) Circ Cardiovasc Genet 10:e001666 |
| Thr363Arg | c.1088C>G | DCM | 3.94 x 10^-5^ | 25163546 | Hass (2015) Eur Heart J 36:18 |
| Pro371Leu | c.1112C>T | HCM | 6.85 x 10^-5^ | 27532257 | Walsh (2017) Genet Med 19:192 |
| Lys400Glu | c.1198A>G | DCM | Absent | 31983221 | Mazzarotto (2020) Circulation 141:387 |
| Glu430Asp | c.1290A>C | LVNC | Absent | 31918855 | Liu (2020) Int J Cardiol 302:117 |
| Gly456Arg | c.1366G>A | HCM | 7.13 x 10^-6^ | 27532257 | Walsh (2017) Genet Med 19:192 |
| Lys466Gln | c.1396A>C | LVNC | Absent | 30471092 | Richard (2019) Clin Genet 95:356 |
| Ala472Gly | c.1415C>G | LVNC | 4.42 x 10^-5^ | 28798025 | Miszalski-Jamka (2017) Circ Cardiovasc Genet 10:e001763 |
| Ala476Pro | c.1426G>C | HCM | Absent | 34598319 | Filatova (2021) Mol Genet Genomic Med 9: |
| Glu485Lys | c.1453G>A | HCM | 5.35 x 10^-5^ | 27532257 | Walsh (2017) Genet Med 19:192 |
| Ala486Gly | c.1457C>G | HCM | Absent | 27532257 | Walsh (2017) Genet Med 19:192 |
| Ile547Thr | c.1640T>C | DCM | 4.99 x 10^-5^ | 28333919 | Bagnall (2017) Genet Med 19:1127 |
| Pro611Thr | c.1831C>A | DCM | Absent | 19881492 | Hassel (2009) Nat Med 15:1281 |
| Arg633Lys | c.1898G>A | DCM | 8.07 x 10^-6^ | 31983221 | Mazzarotto (2020) Circulation 141:387 |
| Pro646Gln | c.1937C>A | HCM | Absent | 27532257 | Walsh (2017) Genet Med 19:192 |
| Tyr652Cys | c.1955A>G | DCM | 7.86 x 10^-5^ | 19881492 | Hassel (2009) Nat Med 15:1281 |
| Thr666Ala | c.1996A>G | DCM | 3.22 x 10^-5^ | 27532257 | Walsh (2017) Genet Med 19:192 |
| Ile671Thr | c.2012T>C | LVNC | 2.87 x 10^-5^ | 30471092 | Richard (2019) Clin Genet 95:356 |
| Ser673Asn | c.2018G>A | LVNC | Absent | 28798025 | Miszalski-Jamka (2017) Circ Cardiovasc Genet 10:e001763 |

**Supplementary Table 9: *DES* missense variants reported in different types of cardiomyopathies**

Abbreviations: DCM, dilated cardiomyopathy; HCM, hypertrophic cardiomyopathy; LVNC, left ventricular noncompaction; RCM, restrictive cardiomyopathy; ACM, arrhythmogenic cardiomyopathy; DRM, desmin-related cardiomyopathy *variants with a Minor Allele Frequency (MAF) > 1 x 10^−4^. MAF retrieved from the Genome Aggregation Database (gnomAD).

| **Desmin Missense Variants** | **Nucleotide Change** | **Disease-associated** | **MAF on GnomAD** | **Pubmed ID** | **Reference** |
| --- | --- | --- | --- | --- | --- |
| Phe24Leu | c.72C>A | ACM | Absent | 27532257 | Walsh (2017) Genet Med 19:192 |
| Ser28Phe | c.83C>T | DCM | Absent | 34011823 | Nguyen (2021) Circ J epub:epub |
| Lys43Glu | c.127A>G | DCM | Absent | 27532257 | Walsh (2017) Genet Med 19:192 |
| Gly65Ser | c.193G>A | DCM | 2.42 x 10^-5^ | 27532257 | Walsh (2017) Genet Med 19:192 |
| Leu69Pro | c.206T>C | LVNC | 1.04 x 10^-5^ | 28798025 | Miszalski-Jamka (2017) Circ Cardiovasc Genet 10:e001763 |
| Glu108Lys | c.322G>A | DCM | Absent | 17325244 | Taylor (2007) Circulation 115:1244 |
| Leu115Ile | c.343C>A | DCM | Absent | 29892087 | Horvat (2019) Genet Med 21:133 |
| Asn116Ser | c.347A>G | ACM | Absent | 20829228 | Klauke (2010) Hum Mol Genet 19:4595 |
| Tyr122Cys | c.365A>G | ACM | Absent | 27532257 | Walsh (2017) Genet Med 19:192 |
| Tyr122His | c.364T>C | RCM | Absent | 31718026 | Brodehl (2019) Genes (Basel) 10:918 |
| Val126Leu | c.376G>T | DCM | Absent | 25163546 | Haas (2015) Eur Heart J 36:1123 |
| Leu136His | c.407T>A | DCM | 7.04 x 10^-5^ | 26265630 | Wilson (2015) Circ Res 117:603 |
| Leu136Pro | c.407T>C | DCM | Absent | 26724190 | Brodehl (2015) J Mol Cell Cardiol 91:207 |
| Glu139Gln | c.415G>C | DCM | 5.57 x 10^-6^ | 31514951 | Gigli (2019) J Am Coll Cardiol 74:1480 |
| Arg150Pro | c.449G>C | DCM | Absent | 27532257 | Walsh (2017) Genet Med 19:192 |
| Lys201Asn | c.603G>C | DCM | 7.07 x 10^-6^ | 28416588 | Dal Ferro (2017) Heart 103:1704 |
| **Arg212Gln* | *c.635G>A* | *LVNC* | *2.19 x 10^-4^* | *28798025* | *Miszalski-Jamka (2017) Circ Cardiovasc Genet 10:e001763* |
| Thr219Pro | c.655A>C | HCM | Absent | 29167554 | Harada (2018) J Hum Genet 63:249 |
| **Arg222His* | *c.665G>A* | *DCM* | *4.00 x 10^-4^* | *28416588* | *Dal Ferro (2017) Heart 103:1704* |
| Arg227Cys | c.679C>T | DCM | 7.95 x 10^-6^ | 28171858 | Yu (2017) Cardiology 137:78 |
| Leu232Phe | c.694C>T | DCM | 1.59 x 10^-5^ | 31983221 | Mazzarotto (2020) Circulation 141:387 |
| Glu234Lys | c.700G>A | HCM | Absent | 28427417 | Chen (2017) J Transl Med 15:78 |
| Val242Glu | c.725T>A | DCM | Absent | 32880476 | Verdonschot (2020) Circ Genom Precis Med 13:476 |
| Arg275Gly | c.823A>G | DCM | Absent | 25163546 | Haas (2015) Eur Heart J 36:1123 |
| Arg278Trp | c.832C>T | DCM | 3.99 x 10^-6^ | 27532257 | Walsh (2017) Genet Med 19:192 |
| Ala285Val | c.854C>T | DCM | Absent | 23300193 | Tse (2013) Hum Mol Genet 22:1395 |
| Ser290Tyr | c.869C>A | DCM | Absent | 31983221 | Mazzarotto (2020) Circulation 141:387 |
| Tyr296Cys | c.887A>G | DCM | Absent | 32041989 | Zhang (2020) Sci Rep 10:2226 |
| Ser298Leu | c.893C>T | DCM | 8.14 x 10^-5^ | 17325244 | Taylor (2007) Circulation 115:1244 |
| **Asp312Asn* | *c.934G>A* | *DCM* | *1.91 x 10^-4^* | *17325244* | *Taylor (2007) Circulation 115:1244* |
| Tyr331Asn | c.991T>A | ACM | Absent | 27532257 | Walsh (2017) Genet Med 19:192 |
| Gly340Asp | c.1019G>A | DCM | Absent | 27532257 | Walsh (2017) Genet Med 19:192 |
| Arg350Trp | c.1048C>T | DCM | 2.39 x 10^-5^ | 17325244 | Taylor (2007) Circulation 115:1244 |
| Ala360Ser | c.1078G>T | LVNC | Absent | 28798025 | Miszalski-Jamka (2017) Circ Cardiovasc Genet 10:e001763 |
| Asp365Glu | c.1095C>A | HCM | Absent | 33297573 | Micheu (2020) Diagnostics (Basel) 10:1061 |
| Arg375Trp | c.1123C>T | DCM | 4.24 x 10^-5^ | 30847666 | van Lint (2019) Neth Heart J 27:304 |
| Arg386His | c.1157G>A | DCM | 3.98 x 10^-6^ | 26458567 | Zhao (2015) Int J Mol Med 36:1479 |
| Leu398Pro | c.1193T>C | LVNC | 3.98 x 10^-6^ | 29447731 | van Waning (2018) J Am Coll Cardiol 71:711 |
| Glu401Asp | c.1203G>C | ACM | Absent | 29212896 | Bermúdez-Jiménez (2017) Circulation 137:1595 |
| Glu401Gly | c.1202A>G | DCM | Absent | 30847666 | van Lint (2019) Neth Heart J 27:304 |
| Ile402Thr | c.1205T>C | DCM | Absent | 33373648 | Fischer (2021) Int J Cardiol 329:167 |
| Ala403Val | c.1208C>T | ACM | Absent | 33232181 | Costa (2021) Circ Genom Precis Med 14:e003047 |
| Arg406Trp | c.1216C>T | DRM | Absent | 11352891 | Wang (2001) Circulation 103(19):2402-7 |
| Leu408Val | c.1222C>G | LVNC | Absent | 29447731 | van Waning (2018) J Am Coll Cardiol 71:711 |
| Glu410Lys | c.1228G>A | ACM | Absent | 33373648 | Fischer (2021) Int J Cardiol 329:167 |
| Glu413Lys | c.1237G>A | RCM | Absent | 16890305 | Pruszczyk (2007) Int J Cardiol 117:244 |
| Arg429Gln | c.1286G>A | ACM | 5.17 x 10^-5^ | 32397162 | Limongelli (2020) Genes (Basel) 11: |
| Arg436Ser | c.1308G>C | DCM | 6.38 x 10^-5^ | 32880476 | Verdonschot (2020) Circ Genom Precis Med 13:476 |
| Ile451Met | c.1353C>G | DCM | 6.45 x 10^-5^ | 10430757 | Li (1999) Circulation 100:461 |
| Thr453Ile | c.1358C>T | RCM | Absent | 16376610 | Arbustini (2006) Eur J Heart Fail 8:477 |
| Arg454Gln | c.1361G>A | DCM | 1.28 x 10^-5^ | 25163546 | Haas (2015) Eur Heart J 36:1123 |
| Gly456Arg | c.1366G>A | HCM | Absent | 32150461 | Hoss (2020) Circ Genom Precis Med 13: |
| *Val459Ile* | *c.1375G>A* | *DCM* | *3.18 x 10^-3^* | *17325244* | *Taylor (2007) Circulation 115:1244* |
| Val469Met | c.1405G>A | HCM | Absent | 16585054 | Muntoni (2006) Brain 129:1260 |

**Supplementary Table 10: *OBSCN* missense variants reported in different types of cardiomyopathies**

Abbreviations: DCM, dilated cardiomyopathy; HCM, hypertrophic cardiomyopathy; ACM, arrhythmogenic cardiomyopathy; MAF: Minor Allelic Frequency.

| **Obscurin Missense Variants** | **Nucleotide Change** | **Disease-associated** | **MAF on GnomAD** | **Pubmed ID** | **Reference** |
| --- | --- | --- | --- | --- | --- |
| Glu963Lys | c.2887G>A | DCM | Absent | 26406308 | Marston (2015) PLoS One 10:e0138568 |
| Arg1221Gln | c.3662G>A | HCM | 7.12 x 10^-5^ | 33658040 | Chung (2021) J Cardiovasc Magn Reson 23:18 |
| Pro2014Gln | c.6041C>A | HCM | Absent | 33658040 | Chung (2021) J Cardiovasc Magn Reson 23:18 |
| Val2161Asp | c.6482T>A | DCM | Absent | 26406308 | Marston (2015) PLoS One 10:e0138568 |
| Phe2809Val | c.8425T>G | DCM | Absent | 26406308 | Marston (2015) PLoS One 10:e0138568 |
| Pro3742Leu | c.11225C>T | HCM | Absent | 33658040 | Chung (2021) J Cardiovasc Magn Reson 23:18 |
| Asp4226Tyr | c.12676G>T | HCM | Absent | 33658040 | Chung (2021) J Cardiovasc Magn Reson 23:18 |
| Gly4287Val | c.12860G>T | DCM | Absent | 32746448 | Burstein (2021) Pediatr Res 89:1470 |
| Arg4344Gln | c.13031G>A | HCM | Absent | 17716621 | Arimura (2007) Biochem Biophys Res Commun 362:281 |
| Ala4484Thr | c.13450G>A | HCM | Absent | 17716621 | Arimura (2007) Biochem Biophys Res Commun 362:281 |
| Arg4856His | c.14582G>A | DCM | Absent | 26406308 | Marston (2015) PLoS One 10:e0138568 |
| Val5196Ile | c.15586G>A | HCM | Absent | 33658040 | Chung (2021) J Cardiovasc Magn Reson 23:18 |
| Arg5215His | c.15644G>A | HCM | Absent | 26573135 | Xu (2015) Sci Rep 17;5:16609 |
| Thr5569Ile | c.16706C>T | HCM | Absent | 33658040 | Chung (2021) J Cardiovasc Magn Reson 23:18 |
| Asp5762Asn | c.17284G>A | HCM | Absent | 33658040 | Chung (2021) J Cardiovasc Magn Reson 23:18 |
| Ala5791Pro | c.17371G>C | ACM | Absent | 28750076 | Forleo (2017) PLoS One 12:e0181842 |
| Arg5852Gln | c.17555G>A | HCM | Absent | 33658040 | Chung (2021) J Cardiovasc Magn Reson 23:18 |
| Asp5966Asn | c.17896G>A | DCM | Absent | 26406308 | Marston (2015) PLoS One 10:e0138568 |
| Gly7500Arg | c.22498G>C | HCM | Absent | 26573135 | Xu (2015) Sci Rep 17;5:16609 |
